# Phylogeographic divergence in the widespread delicate skink (*Lampropholis delicata*) corresponds to dry habitat barriers in eastern Australia

**DOI:** 10.1186/1471-2148-11-191

**Published:** 2011-07-04

**Authors:** David G Chapple, Conrad J Hoskin, Stephanie NJ Chapple, Michael B Thompson

**Affiliations:** 1 School of Biological Sciences, Monash University, Clayton, Victoria 3800, Australia; 2 Museum Victoria, Division of Sciences, GPO Box 666, Melbourne, Victoria 3001, Australia; 3 Allan Wilson Centre for Molecular Ecology and Evolution, School of Biological Sciences, Victoria University of Wellington, PO Box 600, Wellington 6140, New Zealand; 4 Division of Evolution, Ecology & Genetics, Research School of Biology, Australian National University, Canberra, Australian Capital Territory 0200, Australia; 5 School of Marine and Tropical Biology, James Cook University, Townsville, Queensland 4811, Australia; 6 Department of Zoology, University of Melbourne, Melbourne, Victoria 3010, Australia; 7 School of Biological Sciences, University of Sydney, The Heydon-Laurence Building A08, New South Wales 2006, Australia

## Abstract

**Background:**

The mesic habitats of eastern Australia harbour a highly diverse fauna. We examined the impact of climatic oscillations and recognised biogeographic barriers on the evolutionary history of the delicate skink (*Lampropholis delicata*), a species that occurs in moist habitats throughout eastern Australia. The delicate skink is a common and widespread species whose distribution spans 26° of latitude and nine major biogeographic barriers in eastern Australia. Sequence data were obtained from four mitochondrial genes (*ND2, ND4, 12SrRNA, 16SrRNA*) for 238 individuals from 120 populations across the entire native distribution of the species. The evolutionary history and diversification of the delicate skink was investigated using a range of phylogenetic (Maximum Likelihood, Bayesian) and phylogeographic analyses (genetic diversity, Φ_ST_, AMOVA, Tajima's *D*, Fu's *F *statistic).

**Results:**

Nine geographically structured, genetically divergent clades were identified within the delicate skink. The main clades diverged during the late Miocene-Pliocene, coinciding with the decline and fragmentation of rainforest and other wet forest habitats in eastern Australia. Most of the phylogeographic breaks within the delicate skink were concordant with dry habitat or high elevation barriers, including several recognised biogeographic barriers in eastern Australia (Burdekin Gap, St Lawrence Gap, McPherson Range, Hunter Valley, southern New South Wales). Genetically divergent populations were also located in high elevation topographic isolates inland from the main range of *L. delicata *(Kroombit Tops, Blackdown Tablelands, Coolah Tops). The species colonised South Australia from southern New South Wales via an inland route, possibly along the Murray River system. There is evidence for recent expansion of the species range across eastern Victoria and into Tasmania, via the Bassian Isthmus, during the late Pleistocene.

**Conclusions:**

The delicate skink is a single widespread, but genetically variable, species. This study provides the first detailed phylogeographic investigation of a widespread species whose distribution spans virtually all of the major biogeographic barriers in eastern Australia.

## Background

**Figure 1:**
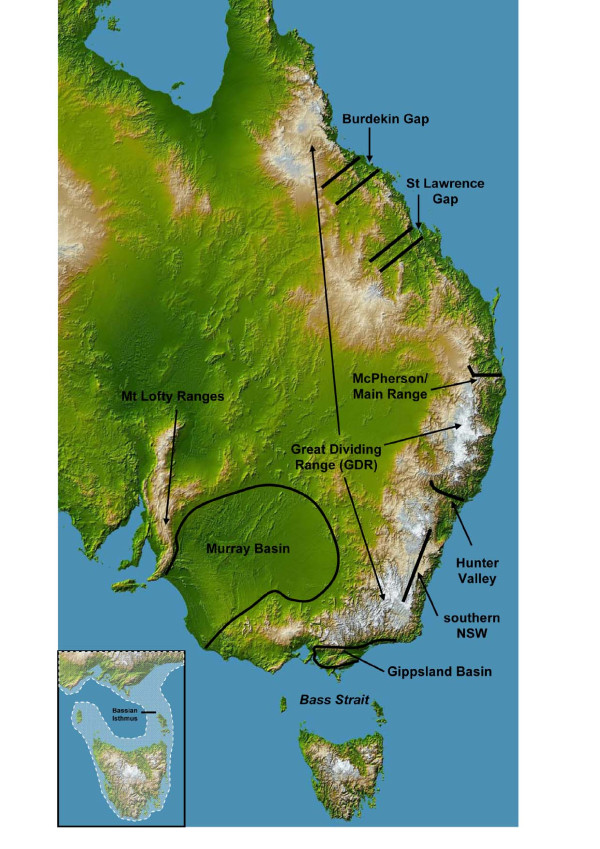
**The major biogeographic barriers in eastern Australia**. A description of each barrier is provided in Table 1. The location of Kroombit Tops is shown in Figure 2. Inset: The coastline of the Bass Strait region 14 kya. Tasmania has repeatedly been connected to the mainland during the Pleistocene by land bridges, with the most recent connection occurring 43-14 kya during the last glacial maxima. The western land bridge was severed 17.5 kya, with the eastern connection (the Bassian Isthmus) being inundated 13 kya, isolating Tasmania from the mainland (after [16]).

The coastal regions of eastern Australia are currently dominated by wet forest and drier sclerophyllous habitats that harbour a highly diverse fauna [1,2]. While the majority (~70%) of the Australian continent is covered by arid or semi-arid vegetation, eastern Australia provides a narrow, but largely continuous expanse of habitat for mesic-adapted species [1,3,4]. These mesic habitats are generated through the presence of the Great Dividing Range (GDR), which abuts the entire length of the east coast (~2,500 km) in a north-south alignment ([5-7]; Figure 1). In the context of an expansive continent that is characterised by low topographic relief, the moderate elevation (~1000-1300 m, maximum ~2300 m) provided by the GDR generates altitudinal, climatic and environmental variation, and precipitates the required moisture to support mesic vegetation [3,5,7].

Although widespread glaciation never occurred in Australia [8,9], climatic oscillations have driven repeated altitudinal and distributional shifts in mesic habitats along the eastern margin of the continent (reviewed in [4]). Palaeoclimatic studies indicate that the extent and composition of the vegetation has fluctuated dramatically over the last 10 myr, although there has been a general transition from rainforest towards drier environments and sclerophyllous vegetation [1,3,8]. The rainforests that had previously dominated eastern Australia contracted between the mid- and late-Miocene, giving way to woodland and open forest vegetation that was more suited to the drier climates [3,10-12]. Lowered sea level associated with globally drier conditions facilitated the expansion of vegetation into the low lying regions of south-eastern Australia (e.g. Gippsland Basin, Murray Basin; Figure 1) that had previously been subject to marine inundation [3,11,12]. Although the extent of rainforests briefly expanded again during the early Pliocene due to a temporary return to warm and wet conditions, by the end of the Pliocene open woodlands, sclerophyllous forests and grasslands dominated the landscape of eastern Australia [3,6,8,10].

The cool-dry to warm-wet climatic fluctuations that commenced during the Pliocene intensified throughout the Pleistocene and led to the repeated expansion and contraction of mesic habitats in eastern Australia and the regular encroachment of drier habitats into the coastal fringes [4,8,13,14]. There was periodic flooding of the low lying coastal and inland basins in eastern Australia during the sea level changes associated with these climatic cycles [3,6,15], which also resulted in the connection of Tasmania (TAS) to the mainland during glacial periods by Bass Strait land bridges [16] (Figure 1). At present, the once widespread rainforest and wet forest vegetation is restricted to small, scattered remnants within a mosaic of dry sclerophyll woodlands and open forests along the east coast [1,17].

The evolutionary history of the resident fauna of the narrow mesic strip along the east coast has been influenced by both habitat barriers and physical barriers (e.g. mountain ranges, sea straits), which led to genetic divergence and, in some cases, speciation of allopatric populations [1,18]. The most well-studied barrier in eastern Australia has been the Black Mountain Corridor (BMC) in the Wet Tropics region of north Queensland (QLD). This thin strip of rainforest currently connects the northern and southern rainforest block of the Wet Tropics but was repeatedly severed in the past by dry forest habitats during globally drier climates [18,19]. Intensive research has revealed largely concordant patterns of genetic divergence across the barrier in a wide range of rainforest taxa [e.g. [19-24]], and improved our understanding of how these barriers, in concert with climatic oscillations, have generated the high levels of biodiversity evident in eastern Australia [18,25]. However, at least nine other biogeographic barriers have been identified in eastern Australia (Tables 1 and 2; Figure 1), several of which have yet to be investigated in detail. These include dry habitat barriers (Burdekin Gap, St Lawrence Gap, Hunter Valley), mountain ranges that act as topographic barriers (McPherson Range, southern New South Wales [NSW]), disjunct inland mountains (Kroombit Tops), sea straits (Bass Strait), and marine basins (Gippsland Basin, Murray Basin) (Tables 1 and 2; Figure 1).

**Table 1:** Description of the recognised biogeographic barriers in eastern Australia (see Figure 1).

Barrier	Explanation of Barrier
Burdekin Gap	A broad region of dry woodland and savanna that extends to the coast and delineates the boundary between the northern rainforests and the mid-eastern Queensland forests [93-95]
St Lawrence Gap	A dry habitat corridor that separates the mid-eastern Queensland forests from the south-eastern Queensland forests [93-95]
Kroombit Tops	A disjunct inland region of high elevation moist habitat that is surrounded by drier eucalypt woodland. An inland cool and wet refuge for rainforest and wet forest adapted species [70,71]
McPherson Range	An east-west spur of the predominately north-south Great Dividing Range that runs along the Queensland/New South Wales border. A montane block of wet forest that represents a hybrid zone for birds and a barrier for lowland and dry forest plant species [93-96]
Hunter Valley	A dry, open, lowland river valley that delineates the southern limit of the eastern biogeographic region and the northern limit of the south-east forest region [93-96]
Southern NSW	Transition from the lowland coastal region to the higher elevation southern highlands region of the Great Dividing Range in New South Wales [71,74]
East Gippsland	Low lying coastal region that has been subject to repeated marine incursion (i.e. Gippsland Basin); abutted to the north by higher elevation regions of the Great Dividing Range [16,97]
Bass Strait	The shallow sea strait (depth 50-80 m, width 240 km) that separates Tasmania from mainland Australia. Land bridges have periodically connected the two landmasses during Pleistocene glacial periods (see Figure 1), with the last connection severed 13 kya [16]
Murray Basin	Low lying region that has been subject to repeated marine incursion (i.e. Murray Basin), bordered to the west by the Mt Lofty Ranges, a known refugia [92,93,97]

The Black Mountain Corridor in the Wet Tropics of north Queensland is not included as the distribution of the delicate skink (*Lampropholis delicata*) does not span this biogeographic barrier.

**Table 2:** The impact of the recognised biogeographic barriers in eastern Australia (Figure 1, Table 1) on vertebrates, invertebrates and plants.

Taxa	Burdekin Gap	St Lawrence Gap	Kroombit Tops	McPherson Range	Hunter Valley	Southern NSW	East Gippsland	Bass Strait	Murray Basin
**Vertebrates**									
Freshwater fish									
*Pseudomugil signifier *[69]	Y	N	-	S	N	-	-	-	-
Amphibians									
*Crinia signifera *[79]	-	-	S	S	N	Y	Y	Y	Y
*Limnodynastes peronii *[67]	Y	N	S	N	Y	S	S	N	-
*Limnodynastes tasmaniensis *[67]	Y	N	S	N	S	S	Y	N	Y
*Litoria aurea *[98]	-	-	-	-	N	-	-	-	-
*Litoria citropa *species group [73,77]	-	-	Y	Y	Y	Y	-	-	-
*Litoria fallax *[66]	Y	S	Y	Y	-	-	-	-	-
Reptiles									
*Acritoscincus duperreyi *[76]	-	-	-	-	-	-	S	Y	Y
*Acritoscincus platynotum *[76]	-	-	-	-	Y	S	-	-	-
*Carlia rubrigularis/rhomboidalis *[22]	Y	-	-	-	-	-	-	-	-
*Diporiphora australis *[68]	Y	Y	S	N	-	-	-	-	-
*Hoplocephalus stephensi *[78]	-	-	S	Y	-	-	-	-	-
*Lampropholis guichenoti *[26]	-	-	-	Y	Y	Y	Y	-	Y
*Lerista bougainvilii *[83]	-	-	-	-	S	S	S	Y	N
*Liopholis whitii *[74]	-	-	-	S	Y	Y	Y	N	Y
*Notechis scutatus *[85]	-	-	-	S	S	S	S	N	Y
*Saproscincus mustelinus *[75]	-	-	-	-	Y	S	S	-	-
Birds									
*Ptilonorhynchus violaceus *[71]	S	S	Y	N	N	Y	N	-	-
*Sericornis frontalis *[19]	N	N	S	S	S	S	S	-	S
*Sericornis magnirostris *[19]	Y	N	S	S	S	-	-	-	-
*Sericornis citeogularis *[19]	Y	-	S	S	S	-	-	-	-
Mammals									
*Dasyurus maculates *[84]	N	N	-	S	N	S	S	Y	-
*Petaurus australis *[99]	Y	S	S	N	S	S	S	-	-
**Invertebrates**									
*Catomerus polymerus *(M) [88]	-	-	-	-	-	-	-	Y	-
*Catostylus mosaicus *(M) [86]	-	-	-	-	-	-	-	Y	-
*Drosophila birchii *[100]	N	S	-	-	-	-	-	-	-
*Nerita atramentosa *(M) [87]	-	-	-	-	-	-	-	Y	-
**Plants**									
*Eucalyptus grandis *[101]	S	S	-	N	S	-	-	-	-

Impact codes: Y = genetic break present across barrier; N: no genetic break observed across the barrier; S = insufficient sampling to examine the impact of the barrier;-= species distribution does not span the barrier. Several marine species (M) are included to investigate the impact of Bass Strait land bridges.

**Figure 2:**
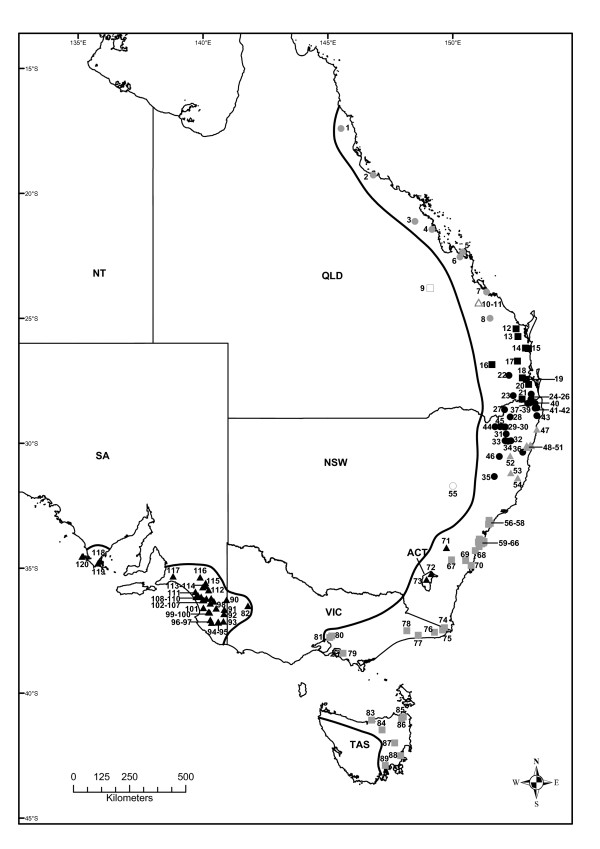
**Map of eastern Australia indicating the collection localities of the *Lampropholis delicata *samples**. The distribution of the nine major clades are indicated: clade 1 (grey solid circles), clade 2 (hollow triangles), clade 3 (black solid squares), clade 4 (black solid circles), clade 5 (grey solid triangles), clade 6 (hollow squares), clade 7 (black solid triangles), clade 8 (hollow circles), clade 9 (grey solid squares). The approximate distribution (solid line) of *L. delicata *is indicated (adapted from [28]), with the population numbers from Additional files 1 and 2 are presented next to the sampling localities.

**Figure 3:**
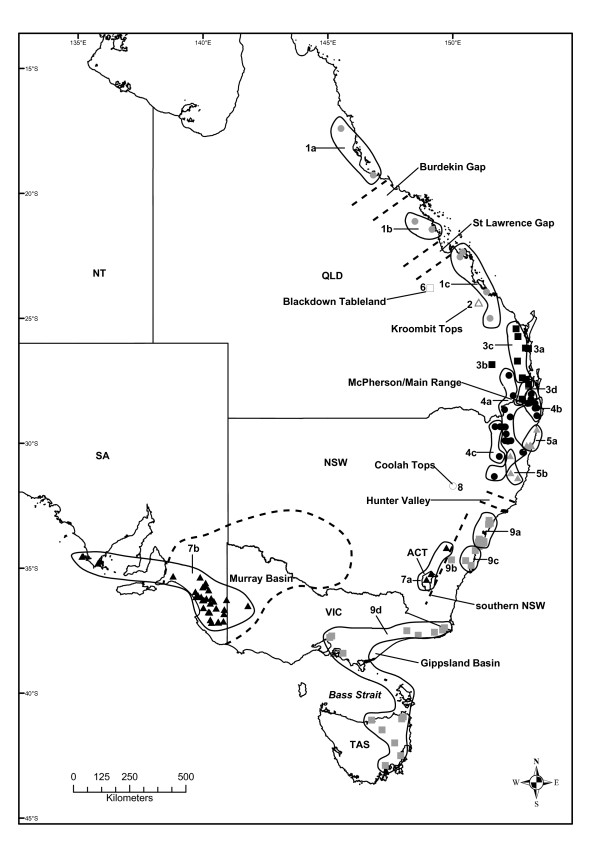
**The distribution of *Lampropholis delicata *clades and subclades**. The distribution of each subclade identified in Figure 4 and 5 is presented (solid lines), along with the location of recognised biogeographic barriers in eastern Australia (dashed lines; Figure 1, Additional file 2).

A recent study investigated the impact of five biogeographic barriers in south-eastern Australia [26]; however, here we adopt a broader approach and examine the influence of nine biogeographic barriers (Tables 1 and 2) throughout eastern Australia on the evolutionary history of the resident biota. In particular, we focus on the delicate skink, *Lampropholis delicata *(De Vis, 1888 [27]), which is unusual in that its distribution is so broad that it spans all of these barriers in eastern Australia (Figure 2, 3). The delicate skink is a small lizard (adult snout-vent length 35-51 mm) whose distribution extends across 26° of latitude from Cairns in north QLD to Hobart in TAS, with disjunct populations in far western Victoria (VIC) and south-eastern South Australia (SA) ([28]; Figure 2, 3). It is a common species that occurs across a range of moist habitats, including rainforests, wet sclerophyll forests, woodland and heaths [28]. However, it also thrives in disturbed habitats and is one the most common skink species in suburban gardens along the east coast [28].

Here we examine the phylogeography of the delicate skink using 2426 bp of mitochondrial DNA sequence data (*ND2, ND4, 12SrRNA, 16SrRNA*) from across the entire native range of the species (Figure 2, 3). Due to its presence in TAS and eastern VIC, Rawlinson [29] suggested that the delicate skink was a glacial relic that had occurred in southern Australia for a prolonged period of time. However, it has been implied that the delicate skink might not be native to TAS, instead reaching the state via human-assisted colonisation. This is because the delicate skink was not detected in TAS until 1963, although subsequent examination of museum collections revealed that previously mis-identified specimens had been collected during the 1920s and 1930s [30]. Unlike many reptile species whose distribution spans Bass Strait, the delicate skink does not occur on Wilsons Promontory (the most southerly projection of the Australian mainland), but it does occur on a Bass Strait Island (Flinders Island) that formed part of the Bassian Isthmus during the last glacial maxima ([28]; Figure 1, 2). We conduct a range of phylogeographic analyses to examine Rawlinson's [29] hypothesis, determine the status of the Tasmanian population, and investigate the impact of historical processes on the evolutionary history of the delicate skink.

## Methods

### Sampling

We obtained tissue samples from 238 *Lampropholis delicata*, representing 120 different populations, from across the entire Australian range of the species (Figure 2, 3; Additional files 1, 2). Samples were obtained from the frozen-tissue collections of several Australian Museums (Australian Museum, South Australian Museum, CSIRO Australian National Wildlife Collection), along with our own field collections (Additional files 1, 2). We included the closely related *L. guichenoti *(Australian Museum NR2639) and an Australian *Eugongylus*-lineage skink *Niveoscincus pretiosus *(Australian Museum NR391) as outgroups in our study.

### DNA extraction, amplification and sequencing

Total genomic DNA was extracted from liver, muscle, toe or tail-tip samples using a Qiagen DNeasy Blood and Tissue Extraction Kit (Qiagen, Hilden, Germany). For each sample we sequenced portions of four mitochondrial genes: *ND2 *(~600 bp), *ND4 *(~700 bp), *12SrRNA *(~700 bp), and *16SrRNA *(~500 bp). These regions were targeted because work across several taxonomic levels in squamate reptiles has indicated useful levels of variability [e.g. [26,31-34]]. The primers used to amplify and sequence these regions are provided in Additional file 3. PCR was conducted as outlined in Greaves *et al*. [35], except on a Corbett Research GC1-960 thermal cycler. PCR products were purified using ExoSAP-IT (USB Corporation, Cleveland, Ohio USA). The purified product was sequenced directly using a BigDye Terminator v3.1 Cycle Sequencing Kit (Applied Biosystems) and then analysed on an ABI 3730XL capillary sequencer.

Sequence data were edited using CONTIGEXPRESS in VECTOR NTI ADVANCE v9.1.0 (Invitrogen), and aligned using the default parameters of CLUSTAL X v1.83[36]. We translated all coding region sequences to confirm that none contained premature stop codons. Sequence data were submitted to GenBank [GenBank:JF438009-JF438959, EF567304, EU567726, EU567768, EU567769, EU567927, EU567928, EU568019, EU568020] (Additional file 1).

### Phylogenetic analyses

Maximum Likelihood (ML) and Bayesian tree building methods were used. We used MODELTEST 3.7 [37] to identify the most appropriate model of sequence evolution based on the AIC criterion. MODELTEST, conducted in PAUP* 4.0b10 [38], was also used to estimate base frequencies, substitution rates, the proportion of invariable sites (I) and the among-site substitution rate variation (G). These values were then used as settings in PhyML 3.0 [39] to generate a ML tree with 500 bootstraps.

MRBAYES 3.1.2 [40] was then used to complete Bayesian analyses. Preliminary analysis of each mtDNA region revealed congruent tree topologies. In order to evaluate partitioning strategies, we used MODELTEST to determine the most appropriate model for each partition. We then conducted a Bayesian analysis for each partitioning strategy, applying the appropriate model of evolution to each partition, and allowing among-partition rate variation. We ran each Bayesian analysis for five million generations, sampling every 100 generations (i.e. 50,000 sampled trees). We ran each analysis twice, using four heated chains per run. We discarded the first 25% of samples as burn-in and the last 37,500 trees were used to estimate the Bayesian posterior probabilities. In order to calculate the AIC and BIC scores for different partitions strategies, we calculated the number of parameters for each. Following McGuire et al. [41], for each parameter we added the number of substitution rates for the model suggested by MODELTEST for that partition (6 for GTR, or 2 for HKY), the number of free equilibrium base frequencies (3 for GTR and HKY), plus one parameter per partition where appropriate for each of I and/or G. For multi-partition strategies, we also added one parameter per partition, corresponding to the among-partition rate multiplier. To calculate the AIC and BIC scores, we used the equations: AIC = -2*L_i _*+ 2*k_i _*and BIC = -2*L_i _*+ (*k_i_*).(ln *n*) (where *L*_i _is the harmonic mean log likelihood for partitioning strategy *i, k_i _*is the total number of parameters for partitioning strategy *i*, and n is the total number of nucleotides). The program TRACER 1.5 [42] was used to check for chain convergence and mixing. Specifically, raw traces of sampled values versus MCMC step numbers were examined to confirm that there was no trend away from the mean and that there were no large fluctuations in the likelihood values.

Bootstrap values (500 ML bootstraps) and Bayesian posterior probabilities were used to assess branch support. We considered branches supported by bootstrap values of 70% or greater [43], and/or posterior probability values greater than or equal to 95% [44] to be supported by our data.

### Molecular diversity and population divergence

Estimates of genetic diversity within *L. delicata *clades (number of haplotypes, *h*; haplotypic diversity, *Hd*; number of polymorphic sites, *S*; nucleotide diversity, π) were calculated in DNASP v4.50 [45]. Tamura-Nei (TrN)-corrected genetic distances within and among clades were calculated in MEGA 4 [46]. Genetic differentiation among clades within *L. delicata *was estimated in ARLEQUIN v3.5 [47]. Pairwise Φ_ST _values (an analogue of Wright's fixation index *F*_ST_) were calculated to estimate among clade differentiation. We conducted hierarchical Analysis of Molecular Variance (AMOVA; [48]) to investigate the impact of the *a priori *(Tables 1 and 2) and *a posteriori *biogeographic barriers on the partitioning of genetic variation within *L. delicata*. Both tests used TrN genetic distances with gamma correction (using the value calculated from MODELTEST). Significance levels of all the estimated values were calculated by 10,000 permutations, and adjusted according to the Bonferroni correction procedure [49] for multiple pairwise comparisons as described by Holm [50].

We used Tajima's *D *[50], Fu's *F *statistic [51] (calculated in ARLEQUIN) and mismatch distributions to test for signatures of population expansion within *L. delicata *clades. Significant and negative Tajima's *D *and Fu's *F *statistic values are indicative of possible population expansion. Mismatch frequency histograms were plotted in DNASP to determine whether the clades exhibited evidence of spatial range expansion or a stationary population history [52]. A smooth bell shape signifies either population expansion or spatial range expansion, whereas a multimodal distribution represents a long history *in situ *[53-56]. To distinguish between these two types of distribution, a raggedness index (RI, sum of the squared difference between neighbouring peaks) and the sum of squared deviations (SSD) between the observed and expected mismatch were calculated using the methods of Schneider & Excoffier [57] in ARLEQUIN. The spatial expansion hypothesis (both RI and SSD) was tested using a parametric bootstrap approach (200 replicates).

As there are no suitable fossil calibration points available for *Lampropholis *skinks, we estimated the divergence time of *L. delicata *clades using an evolutionary rate of 1.3-1.63% sequence divergence per million years, based on mitochondrial DNA calibrations from other squamate reptile groups (1.3%, [58]; 1.42-1.63%, [59]; 1.55%, [60]; 1.62%, [61]; 1.63%, [62]). A strict molecular clock (0.0065-0.00815 per lineage substitution rate), implemented in BEAST v1.6.1 [63], was used to estimate the divergence times within *L. delicata*. The Australian *Eugongylus *lineage is estimated to have originated ~20 mya [64], and this information was used as the maximum age of the tree root. A GTR+I+G model of evolution was employed with a coalescent (Bayesian skyline) tree prior. The analysis was run twice, with 20 million generations per run (total 40 million generations). The output was viewed in TRACER to check that stationarity had been reached, and ensure that the effective sample size (ESS) exceeded 200 [63]. The two separate runs were then combined using LOGCOMBINER v1.6.1, with a maximum clade credibility tree generated in TREEANNOTATOR v1.6.1 and visualised in FIGTREE v1.3.1. A Bayesian skyline plot [65] was also generated in TRACER to examine the magnitude and timing of population size changes in *L. delicata*.

## Results

### Molecular diversity and phylogeographic structure

The edited alignment comprised 2426 characters (550 bp *ND2*, 671 bp *ND4*, 708 bp *12SrRNA*, 497 bp *16SrRNA*; Additional file 4), of which 813 (33.5%) were variable and 587 (24.2%) were parsimony-informative. For the ingroup only, the alignment contained 638 (26.3%) variable characters, of which 543 (22.4%) were parsimony-informative. Base frequencies were unequal (A = 0.3694, T = 0.2172, C = 0.2889, G = 0.1245), but a χ^2 ^test confirmed the homogeneity of base frequencies among sequences (df = 498, *P *= 1.0). The phylogenetic analyses were conducted on a dataset comprising the 165 unique haplotypes that were present within *L. delicata *(Table 3).

**Table 3:** Estimates of genetic diversity for the clades present within *Lampropholis delicata*.

Clade	*n*	*h*	*Hd*	*M*(*S*)	π	Tajima's *D*	*F*s	RI	SSD
**Clade 1**	14	13	0.989	191(187)	0.026	0.378	0.585	0.020	0.033*
1a	2	2	1.0	38(38)	0.016	NA	NA	NA	NA
1b	3	3	1.0	7(7)	0.002	NA	NA	NA	NA
1c	9	8	0.972	19(19)	0.003	-0.305	-2.005	0.063	0.054*
**Clade 2**	8	7	0.893	12(12)	0.001	-1.576*	-2.870*	0.050	0.010
**Clade 3**	19	15	0.965	170(164)	0.022	0.617	2.306	0.023	0.024
3a	4	4	1.0	4(4)	0.001	-0.780	-1.872*	0.222	0.042
3b	3	1	0.0	0(0)	0.000	NA	NA	NA	NA
3c	10	8	0.933	54(53)	0.008	0.017	1.044	0.167	0.053
3d	2	2	1.0	4(4)	0.002	NA	NA	NA	NA
**Clade 4**	48	43	0.996	213(206)	0.017	-0.498	-8.730*	0.002	0.009*
4a	29	24	0.988	119(119)	0.009	-1.006	-3.512	0.005	0.010
4b	14	14	1.0	30(30)	0.003	-1.472	-9.261*	0.015	0.006
4c	5	5	1.0	47(47)	0.011	1.618	0.898	0.280	0.190*
**Clade 5**	18	13	0.948	87(87)	0.013	0.923	1.974	0.027	0.032
5a	11	7	0.873	18(18)	0.002	-1.597	-0.929	0.113	0.036
5b	7	6	0.952	35(35)	0.006	0.344	0.894	0.034	0.023
**Clade 6**	4	2	0.500	1(1)	< 0.001	-0.612	0.172	0.250	0.022
**Clade 7**	55	29	0.937	127(123)	0.005	-1.974*	-4.354	0.026	0.016
7a	3	3	1.0	5(5)	0.001	NA	NA	NA	NA
7b	52	26	0.930	68(68)	0.002	-2.306*	-11.161*	0.033	0.015
**Clade 8**	2	1	0.0	0(0)	0.000	NA	NA	NA	NA
**Clade 9**	70	42	0.943	205(201)	0.023	1.184	2.106	0.011	0.025
9a	24	16	0.913	48(48)	0.005	-0.354	-1.561	0.021	0.022
9b	1	1	NA	NA	NA	NA	NA	NA	NA
9c	9	8	0.972	17(17)	0.003	-0.069	-2.070	0.036	0.020
9d	36	17	0.821	24(24)	0.002	-0.851	-5.160*	0.055	0.038
**Overall**	238	165	0.990	715(625)	0.044				

*n *= sample size, *h *= number of haplotypes, *Hd *= haplotypic diversity, *M *= total number of mutations, *S *= number of segregating (polymorphic) sites, π = nucleotide diversity, *F*s = Fu's *F *statistic, RI = raggedness index, SSD = sum of squared deviations. Asterisks indicate significant Tajima's *D*, Fu's *F *statistic, RI and SSD values.

**Figure 4:**
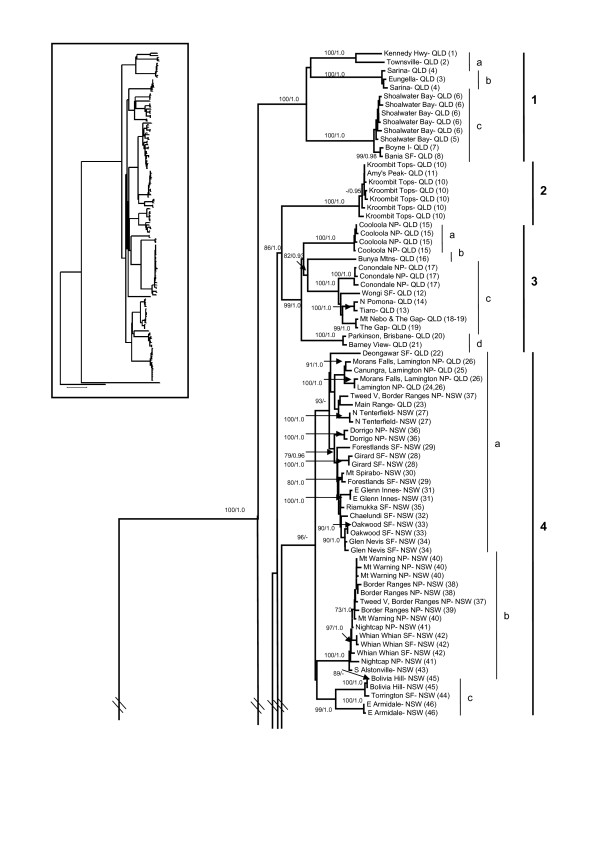
**Phylogram for the delicate skink (*Lampropholis delicata*)**. The phylogeny is based on 2426 bp of mitochondrial DNA (550 bp *ND2*, 671 bp *ND4*, 708 bp *12SrRNA*, 497 bp *16SrRNA*). The population numbers (Figure 2, Additional file 2) are provided in parentheses. The overall tree topology is indicated in the inset. Nine major genetic clades are identified within the delicate skink. Two measures of branch support are indicated with ML bootstraps (500 replicates) on the left and Bayesian posterior probabilities on the right (only values over 70 and 0.9, respectively, are shown).

**Figure 5:**
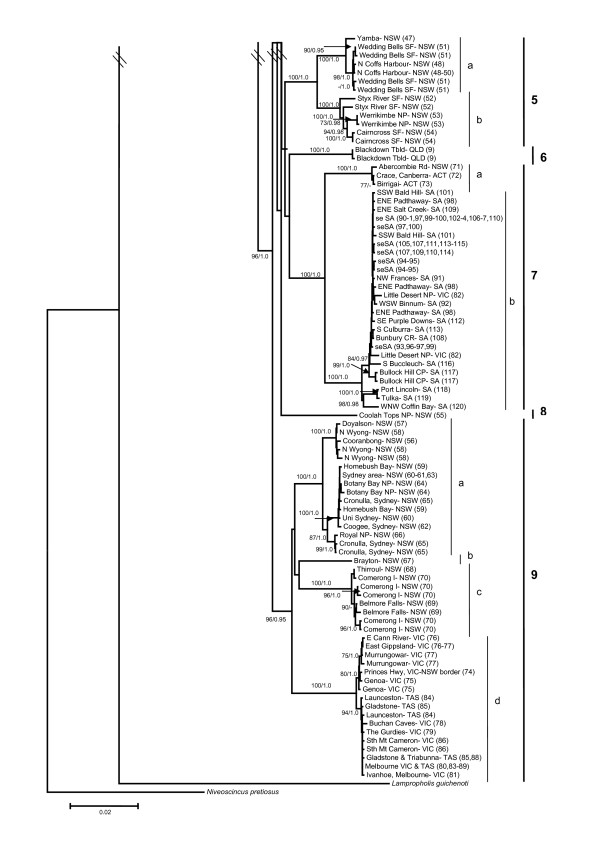
**Continuation of Figure 4**.

The AIC from MODELTEST supported the GTR + I + G substitution model as the most appropriate for our unpartitioned dataset. Parameters estimated under this model were: relative substitution rates (A↔C = 2.1053, A↔G = 44.1898, A↔T = 2.5350, C↔G = 1.1020, C↔T = 29.2135, relative to G↔T = 1.00), proportion of invariable sites (0.5241), and gamma distribution shape parameter (0.7015). We evaluated three partitioning strategies for our dataset (Table 4). The unpartitioned and by gene partitioning strategy were analysed using the GTR + I + G model for all nucletotides. When the data was partitioned by codon, we used a mixture of GTR and HKY models for each partition (Table 4). Both the AIC and BIC scores ranked the most highly parameterised strategy (by gene and codon) as the most appropriate. However, the topologies of the ML, unpartitioned Bayesian and partitioned Bayesian trees were congruent, therefore we present the optimal ML tree (-ln *L *= 14501.43296) with ML bootstrap (BS) values and unpartitioned Bayesian posterior probabilities (PP) indicating branch support (Figure 4, 5). Nine well-supported, non-overlapping clades (labelled from the top to bottom of the tree, and roughly related to their north to south geographic distribution) are present within *L. delicata *(Figure 2, 3, 4, 5), with high levels of haplotypic and nucleotide diversity within each clade (Table 3). The PP's of the main clades and subclades in the partitioned Bayesian analysis were identical to those from the unpartitioned Bayesian analysis presented in Figure 4 and 5, except that the support value for Clade 9 was lower (0.85 rather than 0.95).

**Table 4:** Test of alternative partitioning strategies.

Partition Strategy	No. of Partitions	No. of parameters (*k*_i_)	Harmonic Mean Log-likelihood (*L*_i_)	AIC Score	BIC Score
Unpartitioned	1	11	-15185.35	30392.7	30544.7
By gene	4	48	-14753.32	29602.6	30266.0
By gene and codon	8	82	-14448.71	29061.4*	30194.6*

Modeltest selected the GTR+I+G model for all partitions except for codon position 1 in ND2 and ND4 (HKY+G), ND2 codon position 2 (GTR+I), ND2 codon position 3 and ND2 codon positions 2 & 3 (GTR+G). The asterisk indicates the optimal partitioning strategy.

Clade 1 encompasses populations from coastal northern and eastern QLD, and is comprised of three subclades (Figure 2, 3, 4, 5). Subclade 1a includes populations north of Townsville, subclade 1b contains populations in the Mackay region, and subclade 1c stretches from the Rockhampton region to Bania State Forest (inland from Bundaberg) in south-east QLD (Figure 2, 3, 4, 5). Clade 2 is restricted to Kroombit Tops (Figure 2, 3, 4, 5). A complex mosaic of geographically non-overlapping clades occurs throughout south-eastern QLD and northern NSW (Figure 2, 3, 4, 5). Clade 3 includes populations from the Sunshine Coast and Brisbane region of south-eastern QLD (Figure 2, 3, 4, 5). There is strong support for a close affinity between Clade 2 and Clade 3 (Figure 4, 5). Subclade 3a occurs within Cooloola National Park, while subclade 3b is restricted to the Bunya Mountains (Figure 2, 3, 4, 5). Subclade 3c extends from the Maryborough region to the northern suburbs of Brisbane, with subclade 3d containing populations from the southern suburbs of Brisbane to Barney View on the western side of Lamington National Park (Figure 2, 3, 4, 5).

Clade 4 (96 BS) occurs along the Main Range and throughout the QLD/NSW border region, and in inland northern NSW (Figure 2, 3, 4, 5). Subclade 4a (93 BS) extends from Deongwar State Forest and other areas in south-eastern QLD (Lamington NP, Main Range) through the elevated regions of inland northern NSW to Riamukka State Forest, inland from Port Macquarie (Figure 2, 3, 4, 5). Subclade 4b is restricted to the more coastal regions of northern NSW (Mt Warning NP, Border Ranges NP, Nightcap NP, Whian Whian State Forest, Alstonville), while subclade 4c occurs further inland at Bolivia Hill, Torrington State Forest and near Armidale (Figure 2, 3, 4, 5). Clade 5 extends along the northern NSW coastal region from Yamba to Cairncross State Forest near Port Macquarie, and is comprised of two subclades: subclade 5a (Yamba to Coffs Harbour) and subclade 5b (Styx River State Forest to Cairncross State Forest) (Figure 2, 3, 4, 5). Clade 6 represents a disjunct inland population on the Blackdown Tableland in southern QLD (Figure 2, 3, 4, 5).

Clade 7 is geographically widespread, occurring from the Australian Capital Territory (ACT) and inland southern NSW (subclade 7a) across to western VIC (Little Desert NP) and south-eastern SA (subclade 7b) (Figure 2, 3, 4, 5). Clade 8 is a disjunct population that occurs in Coolah Tops National Park in inland northern NSW (Figure 2, 3, 4, 5). Clade 9 is distributed from the central coast of NSW and throughout eastern VIC and TAS (Figure 2, 3, 4, 5). Subclade 9a encompasses the central coast of NSW and the Sydney region, subclade 9b occurs at Brayton, while subclade 9c comprises populations from coastal southern NSW (Figure 2, 3, 4, 5). Subclade 9d represents a shallow clade that is distributed throughout eastern VIC and TAS (Figure 2, 3, 4, 5).

### Genetic differentiation among clades and divergence time estimates

**Figure 6:**
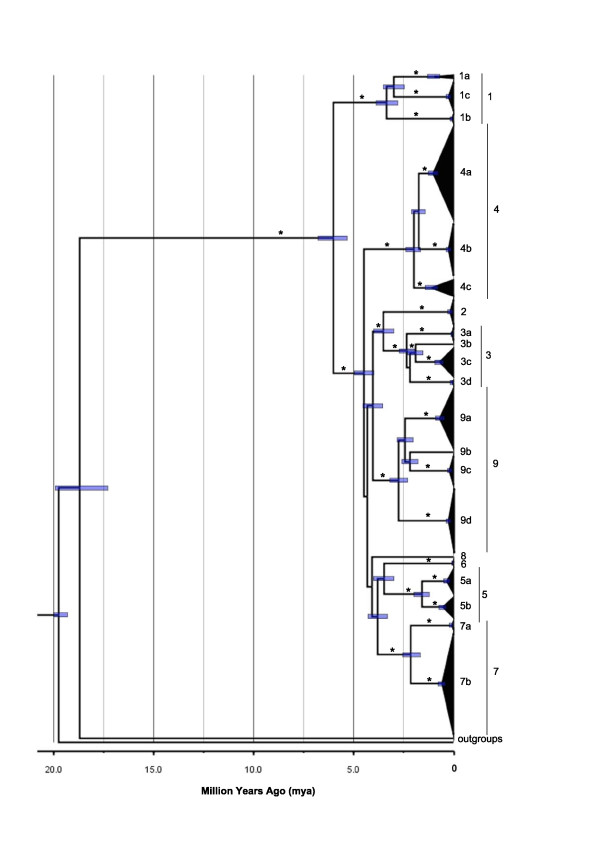
**BEAST maximum clade credibility tree for *Lampropholis delicata***. The divergence times correspond to the mean posterior estimate of their age in millions of years. The bars indicate the 95% HPD interval for the divergence time estimates. Nodes with a posterior probability of > 0.95 are indicated by an asterisk. The clades and subclades identified in Figure 4 and 5 are presented.

Considerable genetic differentiation was evident amongst the nine *L. delicata *clades, with extremely high and statistically significant pairwise Φ_ST _values among clades (Table 5). The only comparisons that were not significant were those involving clades with low sample sizes (e.g. Clade 8). Substantial genetic distances are evident among the clades (4.3-7.4%; Table 5), indicating that the divergences within *L. delicata *occurred during the late Miocene-Pliocene (Figure 6, Additional file 5). The intra-clade genetic divergences in *L. delicata *were 0.0-2.6% (Table 3). The vast majority (97.7%) of genetic variation in *L. delicata *was partitioned among populations (Table 6). The nine *a priori *biogeographic barriers (Tables 1 and 2) accounted for 64.1% of the genetic variation in *L. delicata *(Table 6). This value increased to 66.5% when the two barriers identified *a posteriori *(Blackdown Tableland, Coolah Tops) were included in the analysis (Table 6).

**Table 5:** Mean Tamura-Nei corrected mtDNA genetic distances (below diagonal) and pairwise Φ_ST _(above diagonal) among the major clades (1-9) identified in Figure 4 and 5.

	1	2	3	4	5	6	7	8	9
1	-	0.759*	0.637*	0.698*	0.714*	0.695*	0.858*	0.652	0.637*
2	0.074	-	0.668*	0.721*	0.823*	0.979	0.923*	0.976	0.636*
3	0.071	0.049	-	0.587*	0.604*	0.644*	0.805*	0.608	0.523*
4	0.069	0.051	0.048	-	0.642*	0.676*	0.784*	0.657*	0.581*
5	0.070	0.054	0.047	0.045	-	0.763*	0.851*	0.753	0.588*
6	0.067	0.052	0.051	0.045	0.043	-	0.902*	0.997	0.600*
7	0.074	0.060	0.055	0.052	0.050	0.047	-	0.907*	0.713*
8	0.068	0.052	0.053	0.046	0.048	0.043	0.053	-	0.568*
9	0.070	0.053	0.050	0.050	0.050	0.050	0.054	0.049	-

Asterisks denote statistical significance following Bonferroni correction.

**Table 6:** Hierarchical Analysis of Molecular Variance (AMOVA) for *Lampropholis delicata *populations and biogeographic regions.

Comparison	Observed Partition			Total Variance Components	Total Sum of Squares	*P*
	**Among Populations**	**Within Populations**				
All populations	97.7% (118)	2.3% (119)		70.09	16488.29	< 0.001 (237)
	**Among Regions**	**Among Populations within Regions**	**Within Populations**			
*a priori *biogeographic barriers	64.1% (9)	33.8% (109)	2.1% (119)	78.72	16488.29	All comparisons < 0.001 (237)
*a priori *+ *a posteriori *biogeographic barriers	66.5% (11)	31.4% (107)	2.1% (119)	78.57	16488.29	All comparisons < 0.001 (237)

Separate analyses are conducted for the nine biogeographic barriers that were identified *a priori *(Tables 1 and 2), and including the two additional barriers identified *a posteriori *(i.e. Blackdown Tableland, Coolah Tops). The degrees of freedom are indicated in parentheses. Statistical significance (*P*) was tested with 10,000 permutations.

**Figure 7:**
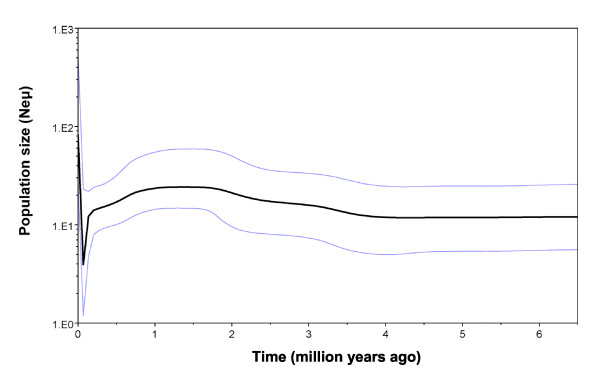
**Bayesian skyline plot for *Lampropholis delicata***. The estimated effective population size over the past 6 myr is presented. The solid line indicates the median population estimate, with the two light blue lines indicating the 95% confidence interval.

The Bayesian skyline plot indicated recent (i.e. last 0.2 myr) contraction then expansion of *L. delicata *populations (Figure 7), although there was no consistent support for the model of spatial expansion in *L. delicata *clades or subclades. Three main clades (2, 4 and 7) and four subclades (3a, 4b, 7b and 9d) deviated significantly from the expectations of neutrality (Tajima's *D*, Fu's *F *statistic) (Table 3), suggesting recent population expansion. However, the RI and SSD values indicated that a model of population expansion could only be conclusively rejected for two main clades (1 and 4) and two subclades (1c and 4c) (Table 3).

## Discussion

The nine main clades of *Lampropholis delicata *appear to have diverged during the late Miocene-Pliocene. Although the current study relied solely on mitochondrial DNA sequence data, the same tree topology is evident in a molecular phylogeny for the *Lampropholis *genus (including representatives from each main *L. delicata *clade) based on mitochondrial DNA and five nuclear genes (C. Hoskin, C. Moritz & D. Chapple, unpublished data). The divergence of *L. delicata *corresponds to a time when rainforest habitat in eastern Australia was in decline as a result of a drying climate, resulting in restriction of rainforest to a series of disjunct remnants that have been described as an 'archipelago of refugia' [1,3,17]. The delicate skink occurs in rainforest or rainforest fringes and therefore likely experienced similar reduction and fragmentation, resulting in genetic divergence among geographically isolated populations. Despite evidence for the expansion and contraction of some clades throughout the Pleistocene, each is geographically structured and non-overlapping (Figure 2, 3). This pattern that has been observed in a range of other taxa (Table 2; [1]), including *L. guichenoti *[26]. Phylogeographic breaks in the delicate skink generally correspond to dry habitat and topographic barriers (i.e. Burdekin Gap, St Lawrence Gap, Kroombit Tops, McPherson Range; Hunter Valley, southern NSW; Figure 1, 2, 3). However, contrary to the hypothesis of Rawlinson [29], the delicate skink appears to be a relatively recent arrival in south-eastern Australia and exhibits no evidence of restricted geneflow across the barriers in this region (e.g. East Gippsland, Bass Strait).

### Phylogeographic structure in the delicate skink corresponds to dry habitat and elevational barriers

Despite its widespread distribution along the east coast of Australia, there is substantial phylogeographic structure across the native range of the delicate skink (Figure 4, 5). In many instances these breaks are concordant with dry habitat corridors, indicating that regions of drier vegetation represent effective barriers to dispersal for the mesic-adapted delicate skink. For instance, the delicate skink exhibits a moderate genetic break (4.5%, subclade 1a vs 1b; Figure 6) across the Burdekin Gap in North QLD. Equivalent Pliocene divergences between populations either side of the Burdekin Gap have been reported in open forest frogs [66,67], rainforest lizards [22], woodland lizards [68], rainforest birds (Pleistocene; [19]) and freshwater fish (Miocene; [69]) (Table 2). In contrast, the St Lawrence Gap north of Rockhampton on the central QLD coast has only been identified as a significant barrier for one lizard species (late Pleistocene-early Pliocene, [68]; Table 2). Although divergence is also evident across the St Lawrence Gap in the delicate skink (4.8%, mid-late Pliocene, subclade 1b vs 1c), a more substantial break is evident a little to the south, between clades 1 and 3 (7.1%, late Miocene) in the Gladstone region (Table 5, Figure 2, 3, 4, 5).

Two high elevation areas (Kroombit Tops, Blackdown Tableland) inland from the main range of *L. delicata *in southern QLD were found to harbour genetically divergent lineages. Both areas are remnant patches of moist forest that are surrounded by drier lowland eucalypt woodland, and are disjunct from the main distribution of the delicate skink along the east coast ([70]; Figure 2, 3). Kroombit Tops (~730 m) was identified *a priori *as a potential habitat isolate for the delicate skink, as the region provides a cooler and wetter refuge for mesic-adapted species ([70,71]; Tables 1 and 2). The Kroombit Tops population of the delicate skink diverged from the surrounding coastal populations during the mid Pliocene (4.9%, Clade 2 vs 3; Table 5, Figure 6), a pattern that has also been observed in a rainforest bird [71], two open forest frogs [66,67], and several open forest reptiles [72] (Table 2). The Blackdown Tablelands are a moderate elevation plateau (~600 m) that provides an isolated refugium for numerous mesic-adapted species. Our results for the delicate skink (4.3-6.7%, Pliocene; Figure 6) provide evidence that the fauna of this region may also be genetically divergent from the coastal populations, a pattern also seen in other open forest reptiles [72].

The Coolah Tops are a high elevation (1200 m) plateau located in inland northern NSW, just to the north of the Hunter Valley (Figure 2, 3). The refugial population of the delicate skink that occurs on the Coolah Tops was found to have diverged from the nearby populations in northern NSW during the early-mid Pliocene (4.6-4.8%; Table 5, Figure 6). The dry habitat in the Hunter River Valley has been demonstrated to represent a major barrier to dispersal in both woodland and wet forest species ([73-76]; Table 2). While the divergence across the Hunter Valley was estimated to have occurred in the Miocene in the congeneric *L. guichenoti*, which is frequently sympatric with *L. delicata *[26], an early-mid Pliocene break was observed across this barrier in the delicate skink (subclade 9a vs Clades 4-5; Figure 2, 3, 4, 5). The divergence estimate for the delicate skink is consistent with those reported for most other species across the Hunter Valley (Table 2).

Our analyses revealed a complex mosaic of geographically structured, non-overlapping clades and subclades (Clades 3-5) in the delicate skink in south-eastern QLD and northern NSW (Figure 2, 3, 4, 5). The McPherson Range that occurs along the border region (Figure 1) is concordant with the phylogeographic break between Clades 3 and 4 (Figure 2, 3). The distribution of Clade 4 extends northwards into south-east QLD to the Main Range, which runs perpendicular to the western edge of the McPherson Range (Figure 1). Similar biogeographic patterns involving the Main and McPherson Ranges occur in wet forest (*Litoria pearsoniana*; [77]) and open forest frogs (*Litoria fallax*; [66]) (Table 2). The early-mid Pliocene split found across the McPherson/Main Ranges in the delicate skink (4.8%; Figure 6) is concordant with that observed in *L. guichenoti*, which also inhabits open woodlands and dry sclerophyll forest [28], but intermediate between that reported for frogs (Miocene; [66,77]) and a wet forest snake (Pleistocene; [78]) (Table 2).

Some relatively minor phylogeographic structure is evident among the populations in the Maryborough, Sunshine Coast and Brisbane regions of south-east QLD (subclades 3a-d; Figure 2, 3, 4, 5). A more substantial break occurs between inland (Clade 4) and coastal (Clade 5) delicate skink populations north of the Hunter Valley in NSW (4.5%, early-mid Pliocene; Table 5, Figure 6). A similar coastal vs inland divergence in northern NSW is shared with White's skink (*Liopholis whitii*, [74]), and reflects an equivalent pattern that is regularly observed in southern NSW (Table 2). Indeed, an analogous pattern is evident within Clade 4 in northern NSW, with subclade 4b occurring near the coastal margin, subclade 4a present in intermediate areas (with secondary contact between 4a and 4b occurring in the Border Ranges NP), and subclade 4c occurring further inland (Figure 2, 3). These biogeographic patterns, combined with the break observed in southern NSW (Figure 2, 3), indicate that high elevation areas may represent barriers to dispersal in the delicate skink.

### The delicate skink is a relatively recent arrival in southern Australia

The five phylogeographic studies that have had sufficient sampling to examine the impact of the elevational and habitat barriers in southern NSW have reported a genetic break between the inland (including the ACT) and coastal regions ([26,71,73,74,79]; Table 2). The impact of this barrier is pronounced in the delicate skink, as populations from the ACT and inland southern NSW are more closely related to the SA populations (3.0%, early Pleistocene-late Pliocene) than the adjacent populations along the NSW coast (5.3%, mid-Pliocene; Figure 2, 3, 4, 5). This indicates that the delicate skink most likely reached SA from the southern NSW region via an inland route, rather than along a coastal dispersal pathway through VIC. The delicate skink may have dispersed through the mesic vegetation that is located along the Murray River, which forms the border between NSW and VIC for the majority of its length (Figure 2, 3). Indeed, the eastern water skink (*Eulamprus quoyii*) has a continuous distribution through the Murray-Darling River system that connects populations along the east coast (North QLD to southern NSW) to an isolated population in south-eastern SA [28]. This biogeographic pattern was not previously suspected for the delicate skink and explains the large distributional gap across western VIC between the eastern suburbs of Melbourne and Little Desert NP in north-western VIC (Figure 2, 3). Given this pattern, it was not possible to examine the impact of the Murray Basin on the delicate skink (Table 2).

Several frog and lizard species exhibit deep phylogeographic breaks in the East Gippsland region [26,67,74,79], a pattern that is believed to be the result of repeated marine inundation of the area since the Miocene (Tables 1 and 2). However, the East Gippsland region does not appear to constitute a barrier to dispersal in the delicate skink, with an extremely shallow clade (intraclade divergence 0.2%) distributed across eastern VIC and across Bass Strait into TAS (Figure 2, 3). The coastal area in East Gippsland has been relatively stable since the late Pleistocene [15,80], enabling the delicate to disperse across eastern VIC from southern NSW.

The delicate skink has colonised TAS during the late Pleistocene, with the presence of shared haplotypes between populations in eastern VIC and TAS indicating a connection between these two regions until relatively recently (~12-15 kya; Table 5, Figure 6, Additional file 2). The timing coincides with the inundation of the Bassian Isthmus, which connected eastern VIC (Wilsons Promotory) to north-eastern TAS between 43-14 kya ([16]; Table 1, Figure 1). Although the delicate skink does not currently occur on Wilsons Promontory, it is present on Flinders Island (which formed part of the Bassian Isthmus) and is common throughout north-eastern TAS ([[28,81], DGC, personal observation]). While a second land bridge was located from western VIC, through the King Island region to western TAS from ~43-17.5 kya [16], the absence of the delicate skink from western VIC would have precluded dispersal of the species via this western route. Fossil evidence for a *Nothofagus *tree species on King Island 38 kya suggests that moist forest vegetation occurred along the Bass Strait land bridges [82], enabling dispersal of the delicate skink into TAS. Although some other species appear not to have used these recent land bridges (frog: *Crinia signigera*, [79]; reptiles: *Acritoscincus duperreyi*, [76]; *Lerista bougainvilli*, [83]; mammals: *Dasyurus maculatus*, [84]), others appear to have dispersed across these Bass Strait land bridges (frogs: *Limnodynastes peronii *and *tasmaniensis*, [67]; reptiles: *Liopholis whitii*, [73]; *Notechis scutatus*, [85]) (Table 2). The repeated presence of the land bridges has also restricted east-west gene flow across Bass Strait in several marine invertebrate species ([86-89]; Table 2).

There is no evidence to support the hypothesis of Rawlinson [29] that the delicate skink is a 'glacial relic' with a relatively long presence in southern Australia. In contrast, our analyses indicate that the delicate skink only colonised VIC and TAS during the late Pleistocene from coastal southern NSW. Although the delicate skink (also known as the rainbow or plague skink in its introduced range) is a successful invasive species in the Hawaiian Islands, New Zealand, and Lord Howe Island [90], there is no strong evidence to suggest that it represents an introduced species in TAS. However, given the relatively shallow genetic divergences within subclade 9d, we are unable to completely exclude the possibility that the delicate skink reached TAS via human-associated colonisation.

## Conclusions

We performed a detailed phylogeographic study of a species found in mesic forests down almost the entire length of eastern Australia. *Lampropholis delicata *is a single widespread, but genetically variable, species consisting of nine geographically structured, non-overlapping clades. This structuring is likely the result of population subdivision across dry habitat barriers (Burdekin Gap, St Lawrence Gap, Hunter Valley), topographic barriers (McPherson Range, southern NSW) and to upland habitat isolates (Kroombit Tops, Blackdown Tableland, Coolah Tops). In contrast, in the south-east of its range, the delicate skink exhibits evidence for recent dispersal into SA via an inland route, and through eastern VIC and across the Bassian Isthmus into TAS. Previous studies have demonstrated geographic variation in morphology, reproductive ecology and life history in the delicate skink [91,92]. Given the presence of multiple divergent lineages across the range, this regional variation in morphology and life history may have a genetic, as well as climatic or environmental, basis.

## Authors' contributions

DGC, MBT and CJH developed the project and obtained funding for the research. The fieldwork and tissue sample collection was conducted by DGC, CJH and SNJC. DGC and SNJC completed the sequencing and analyses. All authors contributed to writing the manuscript, with each reading and approving the final manuscript.

## Supplementary Material

Additional file 1 **Complete collection locality table, with museum specimen and tissue voucher number and GenBank accession numbers**. Click here for file

Additional file 2 **Clades, haplotypes, latitude and longitude for *Lampropholis delicata *populations sampled in the study**. Click here for file

Additional file 3 **Oligonucleotide primers used in this study**. Click here for file

Additional file 4 **The concatenated alignment (fasta format) for the 165 *Lampropholis delicata *haplotypes (*ND2*: 1-550, *ND4*: 551-1221, *12SrRNA*: 1222-1929, *16SrRNA*: 1930-2426)**. Click here for file

Additional file 5 **Divergence time estimates for the main *Lampropholis delicata *clades and subclades**. Click here for file
