# Supplementary material for: Phylogeographic divergence in the widespread delicate skink (Lampropholis delicata) corresponds to dry habitat barriers in eastern Australia
Source: BMC Evol Biol. 2011 Jul 4;11:191. doi: 10.1186/1471-2148-11-191 (PMC3141439; doi:10.1186/1471-2148-11-191)
Supplement: Additional file 1 — Complete collection locality table, with museum specimen and tissue voucher number and GenBank accession numbers. [file 1471-2148-11-191-S1.DOC]

**Additional file 1** Locality data, museum voucher specimen information, and GenBank accession numbers for the *Lampropholis delicata* samples used in this study. State abbreviations: QLD = Queensland; NSW = New South Wales; ACT = Australian Capital Territory; VIC = Victoria; TAS = Tasmania; SA = South Australia. Museum acronyms: ABTC = Australian Biological Tissue Collection (housed at the South Australian Museum); AM = Australian Museum, Sydney; ANWC = CSIRO Australian National Wildlife Collection, Canberra; EBU = Evolutionary Biology Unit, Australian Museum; NMV = Museum Victoria, Melbourne; QM = Queensland Museum, Brisbane; SAM = South Australian Museum, Adelaide.

| Pop. | Collection Locality | State | Sample Code | Museum Voucher | Tissue Code | Clade | GenBank Accession Number | | | |
| --- | --- | --- | --- | --- | --- | --- | --- | --- | --- | --- |
|  |  |  |  |  |  |  | ND2 | ND4 | 12SrRNA | 16SrRNA |
| 1 | 6 km N The Crater turnoff on Kennedy | QLD | LDA65 | SAMAR55858 | ABTC77179 | 1a | JF438213 | JF438450 | JF438688 | JF438926 |
| 2 | Railway estate, Townsville | QLD | LDA2 | ANWC R5114 | ANWC R5114 | 1a | JF438009 | JF438247 | JF438484 | JF438722 |
| 3 | Eungella | QLD | LDA158 | — | ABTC51272 | 1b | JF438079 | JF438316 | JF438554 | JF438792 |
| 4 | near Sarina | QLD | LDA145 | — | ABTC31948 | 1b | JF438065 | JF438302 | JF438540 | JF438778 |
|  |  |  | LDA149 | — | ABTC31947 | 1b | JF438069 | JF438306 | JF438544 | JF438782 |
| 5 | Shoalwater Bay | QLD | LDA147 | QMJ58942 | ABTC31984 | 1c | JF438067 | JF438304 | JF438542 | JF438780 |
| 6 | Shoalwater Bay Army reserve, N Rockhampton | QLD | LDA7 | ANWC R5384 | ANWC R5384 | 1c | JF438014 | JF438252 | JF438489 | JF438727 |
|  |  |  | LDA8 | ANWC R5386 | ANWC R5386 | 1c | JF438015 | JF438253 | JF438490 | JF438728 |
|  |  |  | LDA9 | ANWC R5394 | ANWC R5394 | 1c | JF438016 | JF438254 | JF438491 | JF438729 |
|  |  |  | LDA10 | ANWC R5397 | ANWC R5397 | 1c | JF438017 | JF438255 | JF438492 | JF438730 |
|  |  |  | LDA11 | ANWC R5557 | ANWC R5557 | 1c | JF438027 | JF438265 | JF438502 | JF438740 |
| 7 | Kidman Crt, Boyne Island | QLD | LDA66 | SAMAR55620 | ABTC76910 | 1c | JF438214 | JF438451 | JF438689 | JF438927 |
| 8 | Bania SF | QLD | LDA233 | — | NMVZ10334 | 1c | JF438148 | JF438385 | JF438623 | JF438861 |
|  |  |  | LDA238 | — | NMVZ10338 | 1c | JF438152 | JF438389 | JF438627 | JF438865 |
| 9 | Blackdown Tableland | QLD | LDA232 | — | NMVZ10333 | 6 | JF438147 | JF438384 | JF438622 | JF438860 |
|  |  |  | LDA234 | — | NMVZ10335 | 6 | JF438149 | JF438386 | JF438624 | JF438862 |
|  |  |  | LDA236 | — | NMVZ10336 | 6 | JF438150 | JF438387 | JF438625 | JF438863 |
|  |  |  | LDA237 | — | NMVZ10337 | 6 | JF438151 | JF438388 | JF438626 | JF438864 |
| 10 | Kroombit Tops | QLD | LDA61 | SAMAR55625 | ABTC76914 | 2 | JF438209 | JF438446 | JF438684 | JF438922 |
|  |  |  | LDA63 | SAMAR55624 | ABTC76913 | 2 | JF438211 | JF438448 | JF438686 | JF438924 |
|  |  |  | LDA64 | SAMAR55623 | ABTC76912 | 2 | JF438212 | JF438449 | JF438687 | JF438925 |
|  |  |  | LDA86 | QMJ54882 | ABTC24237 | 2 | JF438233 | JF438470 | JF438708 | JF438946 |
|  |  |  | LDA173 | — | ABTC51298 | 2 | JF438096 | JF438333 | JF438571 | JF438809 |
|  |  |  | LDA175 | — | ABTC51299 | 2 | JF438098 | JF438335 | JF438573 | JF438811 |
|  |  |  | LDA239 | — | NMVZ10339 | 2 | JF438153 | JF438390 | JF438628 | JF438866 |
| 11 | Tablelands Rd, 5km E Amy's Peak, Kroombit Tops | QLD | LDA62 | SAMAR55631 | ABTC76920 | 2 | JF438210 | JF438447 | JF438685 | JF438923 |
| 12 | Wongi SF | QLD | LDA220 | — | NMVZ10325 | 3c | JF438136 | JF438373 | JF438611 | JF438849 |
| 13 | Tiaro, near Gympie | QLD | LDA243 | — | NMVZ10342 | 3c | JF438158 | JF438395 | JF438633 | JF438871 |
| 14 | 25 km N Pomona | QLD | LDA103 | QMJ46098 | ABTC03917 | 3c | JF438020 | JF438258 | JF438495 | JF438733 |
| 15 | Lake Poonah, Cooloola NP | QLD | LDA226 | — | NMVZ10329 | 3a | JF438140 | JF438377 | JF438615 | JF438853 |
|  |  |  | LDA227 | — | — | 3a | JF438141 | JF438378 | JF438616 | JF438854 |
|  |  |  | LDA228 | — | — | 3a | JF438142 | JF438379 | JF438617 | JF438855 |
|  |  |  | LDA229 | — | NMVZ10330 | 3a | JF438143 | JF438380 | JF438618 | JF438856 |
| 16 | Bunya Mountains | QLD | LDA74 | — | ABTC03856 | 3b | JF438222 | JF438459 | JF438697 | JF438935 |
|  |  |  | LDA80 | — | ABTC03932 | 3b | JF438227 | JF438464 | JF438702 | JF438940 |
|  |  |  | LDA84 | — | ABTC03857 | 3b | JF438231 | JF438468 | JF438706 | JF438944 |
| 17 | Booloumba Creek, Conondale NP | QLD | LDA240 | — | NMVZ10340 | 3c | JF438155 | JF438392 | JF438630 | JF438868 |
|  |  |  | LDA241 | — | NMVZ10341 | 3c | JF438156 | JF438393 | JF438631 | JF438869 |
|  |  |  | LDA244 | — | NMVZ10343 | 3c | JF438159 | JF438396 | JF438634 | JF438872 |
| 18 | Mt Nebo, D'Aguilar Range | QLD | LDA219 | — | — | 3c | JF438134 | JF438371 | JF438609 | JF438847 |
| 19 | The Gap, Brisbane | QLD | LDA223 | — | NMVZ10326 | 3c | JF438137 | JF438374 | JF438612 | JF438850 |
|  |  |  | LDA224 | — | NMVZ10327 | 3c | JF438138 | JF438375 | JF438613 | JF438851 |
|  |  |  | LDA225 | — | NMVZ10328 | 3c | JF438139 | JF438376 | JF438614 | JF438852 |
| 20 | Parkinson, southern Brisbane | QLD | LDA242 | — | — | 3d | JF438157 | JF438394 | JF438632 | JF438870 |
| 21 | Barney View | QLD | LDA104 | QMJ46094 | ABTC03929 | 3d | JF438021 | JF438259 | JF438496 | JF438734 |
| 22 | Deongwar SF | QLD | LDA230 | — | NMVZ10331 | 4a | JF438145 | JF438382 | JF438620 | JF438858 |
| 23 | Main Range | QLD | LDA231 | — | NMVZ10332 | 4a | JF438146 | JF438383 | JF438621 | JF438859 |
| 24 | Python Rock, Lamington NP | QLD | LDA245 | — | — | 4a | JF438160 | JF438397 | JF438635 | JF438873 |
| 25 | Canungra Village, Lamington NP | QLD | LDA247 | — | NMVZ10344 | 4a | JF438161 | JF438398 | JF438636 | JF438874 |
| 26 | Morans Falls, Lamington NP | QLD | LDA258 | — | NMVZ10347 | 4a | JF438171 | JF438408 | JF438646 | JF438884 |
|  |  |  | LDA259 | — | NMVZ10348 | 4a | JF438172 | JF438409 | JF438647 | JF438885 |
|  |  |  | LDA260 | — | — | 4a | JF438174 | JF438411 | JF438649 | JF438887 |
| 27 | 50 km N Tenterfield, Wilsons Downfall Rd | NSW | LDA37 | AMR152018 | EBU7553 | 4a | JF438185 | JF438422 | JF438660 | JF438898 |
|  |  |  | LDA38 | AMR152019 | EBU7554 | 4a | JF438186 | JF438423 | JF438661 | JF438899 |
|  |  |  | LDA39 | AMR152020 | EBU7555 | 4a | JF438187 | JF438424 | JF438662 | JF438900 |
| 28 | Girard SF | NSW | LDA40 | AMR152001 | EBU31443 | 4a | JF438188 | JF438425 | JF438663 | JF438901 |
|  |  |  | LDA41 | AMR152002 | EBU31444 | 4a | JF438189 | JF438426 | JF438664 | JF438902 |
| 29 | Forestlands SF, trail off Spirabo FT ~5 km N Gurrs Rd | NSW | LDA48 | AMR157272 | EBU33686 | 4a | JF438196 | JF438433 | JF438671 | JF438909 |
|  |  |  | LDA49 | AMR157273 | EBU33687 | 4a | JF438197 | JF438434 | JF438672 | JF438910 |
| 30 | Mt Spirabo | NSW | LDA100 | — | ABTC25290 | 4a | JF438018 | JF438256 | JF438493 | JF438731 |
| 31 | 45 km E Glen Innes on Glen Innes-Grafton Rd | NSW | LDA46 | AMR157065 | EBU33371 | 4a | JF438194 | JF438431 | JF438669 | JF438907 |
|  |  |  | LDA47 | AMR157066 | EBU33373 | 4a | JF438195 | JF438432 | JF438670 | JF438908 |
| 32 | Chaelundi SF, Calicoe Creek, Liberation FT | NSW | LDA50 | AMR139350 | EBU3598 | 4a | JF438198 | JF438435 | JF438673 | JF438911 |
| 33 | Oakwood SF, Oakwood FT, N London Bridge FT | NSW | LDA51 | AMR139163 | EBU3446 | 4a | JF438199 | JF438436 | JF438674 | JF438912 |
|  |  |  | LDA52 | AMR139164 | EBU3447 | 4a | JF438200 | JF438437 | JF438675 | JF438913 |
|  |  |  | LDA53 | AMR139165 | EBU3448 | 4a | JF438201 | JF438438 | JF438676 | JF438914 |
| 34 | Glen Nevis SF, 3.3 km E Starlight Lookout | NSW | LDA54 | AMR139176 | EBU3444 | 4a | JF438202 | JF438439 | JF438677 | JF438915 |
|  |  |  | LDA55 | AMR139179 | EBU3442 | 4a | JF438203 | JF438440 | JF438678 | JF438916 |
| 35 | Riamukka SF, Grundy Fire Tower Area | NSW | LDA34 | AMR148250 | EBU6040 | 4a | JF438182 | JF438419 | JF438657 | JF438895 |
|  |  |  | LDA35 | AMR148251 | EBU6041 | 4a | JF438183 | JF438420 | JF438658 | JF438896 |
| 36 | Dorrigo NP, Never Never Picnic Area | NSW | LDA23 | AMR138172 | EBU3382 | 4a | JF438144 | JF438381 | JF438619 | JF438857 |
|  |  |  | LDA24 | AMR138173 | EBU3383 | 4a | JF438154 | JF438391 | JF438629 | JF438867 |
| 37 | Border Ranges NP, Tweed Valley Lookout | NSW | LDA18 | AMR133484 | EBU2954 | 4a | JF438103 | JF438340 | JF438578 | JF438816 |
|  |  |  | LDA19 | AMR151816 | EBU7489 | 4b | JF438110 | JF438347 | JF438585 | JF438823 |
|  |  |  | LDA20 | AMR151817 | EBU7490 | 4a | JF438119 | JF438356 | JF438594 | JF438832 |
| 38 | Border Ranges NP, Lophostemon Falls | NSW | LDA16 | AMR133473 | EBU2944 | 4b | JF438081 | JF438318 | JF438556 | JF438794 |
|  |  |  | LDA17 | AMR133474 | EBU2945 | 4b | JF438092 | JF438329 | JF438567 | JF438805 |
| 39 | Border Ranges NP, Pinnacle | NSW | LDA87 | — | ABTC14080 | 4b | JF438234 | JF438471 | JF438709 | JF438947 |
| 40 | Mount Warning NP | NSW | LDA15 | AMR133462 | EBU2936 | 4b | JF438070 | JF438307 | JF438545 | JF438783 |
|  |  |  | LDA81 | — | ABTC14081 | 4b | JF438228 | JF438465 | JF438703 | JF438941 |
|  |  |  | LDA256 | — | NMVZ10345 | 4b | JF438169 | JF438406 | JF438644 | JF438882 |
|  |  |  | LDA257 | — | NMVZ10346 | 4b | JF438170 | JF438407 | JF438645 | JF438883 |
| 41 | Nightcap NP, near Terania Creek Picnic Area | NSW | LDA21 | AMR138076 | EBU3345 | 4b | JF438130 | JF438367 | JF438605 | JF438843 |
|  |  |  | LDA22 | AMR138109 | EBU3353 | 4b | JF438135 | JF438372 | JF438610 | JF438848 |
| 42 | Whian Whian SF | NSW | LDA68 | SAMAR33693 | ABTC03968 | 4b | JF438216 | JF438453 | JF438691 | JF438929 |
|  |  |  | LDA70 | SAMAR33687 | ABTC03967 | 4b | JF438218 | JF438455 | JF438693 | JF438931 |
|  |  |  | LDA71 | SAMAR33686 | ABTC03966 | 4b | JF438219 | JF438456 | JF438694 | JF438932 |
| 43 | Vict. Pk., S Alstonville | NSW | LDA105 | SAMAR33689 | ABTC03970 | 4b | JF438022 | JF438260 | JF438497 | JF438735 |
| 44 | Torrington State Recreational Area, Torrington | NSW | LDA56 | AMR152097 | EBU31562 | 4c | JF438204 | JF438441 | JF438679 | JF438917 |
| 45 | Bolivia Hill | NSW | LDA90 | SAMAR34756 | ABTC16655 | 4c | JF438237 | JF438474 | JF438712 | JF438950 |
|  |  |  | LDA97 | SAMAR34754 | ABTC16653 | 4c | JF438244 | JF438244 | JF438719 | JF438957 |
| 46 | 18.5 km from Armidale on Ebor Rd | NSW | LDA42 | AMR159668 | EBU33992 | 4c | JF438190 | JF438427 | JF438665 | JF438903 |
|  |  |  | LDA43 | AMR159672 | EBU33999 | 4c | JF438191 | JF438428 | JF438666 | JF438904 |
| 47 | Yamba tip | NSW | LDA76 | SAMAR33682 | ABTC03959 | 5a | JF438223 | JF438460 | JF438698 | JF438936 |
| 48 | Nana Creek Area, N Coffs Harbour | NSW | LDA3 | ANWC R5291 | ANWC R5291 | 5a | JF438010 | JF438248 | JF438485 | JF438723 |
|  |  |  | LDA4 | ANWC R5292 | ANWC R5292 | 5a | JF438011 | JF438249 | JF438486 | JF438724 |
|  |  |  | LDA5 | ANWC R5294 | ANWC R5294 | 5a | JF438012 | JF438250 | JF438487 | JF438725 |
| 49 | Glenreagh Area, N Coffs Harbour | NSW | LDA6 | ANWC R5330 | ANWC R5330 | 5a | JF438013 | JF438251 | JF438488 | JF438726 |
| 50 | Madman's Creek | NSW | LDA250 | — | — | 5a | JF438163 | JF438400 | JF438638 | JF438876 |
| 51 | Wedding Bells SF | NSW | LDA251 | — | — | 5a | JF438164 | JF438401 | JF438639 | JF438877 |
|  |  |  | LDA252 | — | — | 5a | JF438165 | JF438402 | JF438640 | JF438878 |
|  |  |  | LDA253 | — | — | 5a | JF438166 | JF438403 | JF438641 | JF438879 |
|  |  |  | LDA254 | — | — | 5a | JF438167 | JF438404 | JF438642 | JF438880 |
|  |  |  | LDA255 | — | — | 5a | JF438168 | JF438405 | JF438643 | JF438881 |
| 52 | Styx River SF, ~7 km on Styx Rd from Pt Lookout Rd | NSW | LDA25 | AMR138202 | EBU4769 | 5b | JF438162 | JF438399 | JF438637 | JF438875 |
|  |  |  | LDA26 | AMR138203 | EBU4770 | 5b | JF438173 | JF438410 | JF438648 | JF438886 |
|  |  |  | LDA27 | AMR138204 | EBU4771 | 5b | JF438175 | JF438412 | JF438650 | JF438888 |
| 53 | Werrikimbe NP, Plateau Beach | NSW | LDA44 | AMR153783 | EBU32585 | 5b | JF438192 | JF438429 | JF438667 | JF438905 |
|  |  |  | LDA45 | AMR153784 | EBU32586 | 5b | JF438193 | JF438430 | JF438668 | JF438906 |
| 54 | Cairncross SF | NSW | LDA73 | SAMAR33690 | ABTC03986 | 5b | JF438221 | JF438458 | JF438696 | JF438934 |
|  |  |  | LDA93 | SAMAR33691 | ABTC03987 | 5b | JF438240 | JF438477 | JF438715 | JF438953 |
| 55 | Coolah Tops NP, Rocky Creek Falls Picnic Area | NSW | LDA57 | AMR152164 | EBU31679 | 8 | JF438205 | JF438442 | JF438680 | JF438918 |
|  |  |  | LDA58 | AMR152179 | — | 8 | JF438206 | JF438443 | JF438681 | JF438919 |
| 56 | Matthew Valley Rd, via Cooranbong | NSW | LDA72 | SAMAR40864 | ABTC12651 | 9a | JF438220 | JF438457 | JF438695 | JF438933 |
| 57 | Doyalson | NSW | LDA69 | AMR104203 | ABTC11139 | 9a | JF438217 | JF438454 | JF438692 | JF438930 |
| 58 | Warnervale Aerodome, N of Wyong | NSW | LDA28 | AMR148102 | EBU5699 | 9a | JF438176 | JF438413 | JF438651 | JF438889 |
|  |  |  | LDA29 | AMR148103 | EBU5700 | 9a | JF438177 | JF438414 | JF438652 | JF438890 |
|  |  |  | LDA30 | AMR148104 | EBU5701 | 9a | JF438178 | JF438415 | JF438653 | JF438891 |
| 59 | Homebush Bay, Cumbungi Wetland | NSW | LDA59 | AMR141001 | EBU4514 | 9a | JF438207 | JF438444 | JF438682 | JF438920 |
|  |  |  | LDA60 | AMR141002 | EBU4515 | 9a | JF438208 | JF438445 | JF438683 | JF438921 |
| 60 | University of Sydney | NSW | LDA106 | — | NMVZ10292 | 9a | JF438023 | JF438261 | JF438498 | JF438736 |
|  |  |  | LDA109 | — | NMVZ10295 | 9a | JF438026 | JF438264 | JF438501 | JF438739 |
|  |  |  | LDA115 | — | NMVZ10301 | 9a | JF438033 | JF438271 | JF438508 | JF438746 |
|  |  |  | LDA116 | — | NMVZ10302 | 9a | JF438034 | JF438272 | JF438509 | JF438747 |
|  |  |  | LDA123 | — | NMVZ10309 | 9a | JF438041 | JF438279 | JF438516 | JF438754 |
| 61 | Padstow, Sydney | NSW | LDA119 | — | NMVZ10305 | 9a | JF438037 | JF438275 | JF438512 | JF438750 |
| 62 | Coogee, Sydney | NSW | LDA77 | SAMAR34891 | ABTC16812 | 9a | JF438224 | JF438461 | JF438699 | JF438937 |
|  |  |  | LDA78 | SAMAR34889 | ABTC16810 | 9a | JF438225 | JF438462 | JF438700 | JF438938 |
|  |  |  | LDA91 | SAMAR34892 | ABTC16813 | 9a | JF438238 | JF438475 | JF438713 | JF438951 |
| 63 | Hurstville, Sydney | NSW | LDA121 | — | NMVZ10307 | 9a | JF438039 | JF438277 | JF438514 | JF438752 |
|  |  |  | LDA122 | — | NMVZ10308 | 9a | JF438040 | JF438278 | JF438515 | JF438753 |
| 64 | Botany Bay NP | NSW | LDA107 | — | NMVZ10293 | 9a | JF438024 | JF438262 | JF438499 | JF438737 |
|  |  |  | LDA108 | — | NMVZ10294 | 9a | JF438025 | JF438263 | JF438500 | JF438738 |
| 65 | Cronulla Sewage Treatment Plant, Kurnell Peninsula | NSW | LDA31 | AMR147719 | EBU6141 | 9a | JF438179 | JF438416 | JF438654 | JF438892 |
|  |  |  | LDA32 | AMR147720 | EBU6142 | 9a | JF438180 | JF438417 | JF438655 | JF438893 |
|  |  |  | LDA33 | AMR148095 | EBU5966 | 9a | JF438181 | JF438418 | JF438656 | JF438894 |
| 66 | Royal NP | NSW | LDA117 | — | NMVZ10303 | 9a | JF438035 | JF438273 | JF438510 | JF438748 |
| 67 | 3 km SW Brayton | NSW | LDA85 | SAMAR39166 | ABTC12413 | 9b | JF438232 | JF438469 | JF438707 | JF438945 |
| 68 | Thirroul, N Side of Seafoam Ave, W of Railway Line | NSW | LDA36 | AMR148555 | EBU6327 | 9c | JF438184 | JF438421 | JF438659 | JF438897 |
| 69 | Rubbish tip, 4km NW Belmore Falls | NSW | LDA79 | SAMAR39156 | ABTC12407 | 9c | JF438226 | JF438463 | JF438701 | JF438939 |
|  |  |  | LDA89 | SAMAR39158 | ABTC12409 | 9c | JF438236 | JF438473 | JF438711 | JF438949 |
| 70 | Comerong Island | NSW | LDA110 | — | NMVZ10296 | 9c | JF438028 | JF438266 | JF438503 | JF438741 |
|  |  |  | LDA111 | — | NMVZ10297 | 9c | JF438029 | JF438267 | JF438504 | JF438742 |
|  |  |  | LDA112 | — | NMVZ10298 | 9c | JF438030 | JF438268 | JF438505 | JF438743 |
|  |  |  | LDA113 | — | NMVZ10299 | 9c | JF438031 | JF438269 | JF438506 | JF438744 |
|  |  |  | LDA114 | — | NMVZ10300 | 9c | JF438032 | JF438270 | JF438507 | JF438745 |
|  |  |  | LDA118 | — | NMVZ10304 | 9c | JF438036 | JF438274 | JF438511 | JF438749 |
| 71 | 2.6 km N Abercombie Rd crossing | NSW | LDA156 | SAMAR40813 | ABTC57500 | 7a | JF438077 | JF438314 | JF438552 | JF438790 |
| 72 | Crace, Canberra | ACT | LDA12 | ANWC R6673 | ANWC R6673 | 7a | JF438038 | JF438276 | JF438513 | JF438751 |
| 73 | Birrigai | ACT | LDA159 | SAMAR22243 | ABTC54232 | 7a | JF438080 | JF438317 | JF438555 | JF438793 |
| 74 | Princes Hwy, last rest stop VIC side VIC/NSW border | VIC | LDA195 | NMVD73630 | NMVZ6228 | 9d | JF438116 | JF438353 | JF438591 | JF438829 |
| 75 | parkland near Genoa River Bridge, Genoa | VIC | LDA193 | — | NMVZ10313 | 9d | JF438114 | JF438351 | JF438589 | JF438827 |
|  |  |  | LDA194 | NMVD73629 | NMVZ6227 | 9d | JF438115 | JF438352 | JF438590 | JF438828 |
| 76 | Drummer Crk Picnic Grd, E Cann River, Princes Hwy | VIC | LDA190 | NMVD73627 | NMVZ6225 | 9d | JF438111 | JF438348 | JF438586 | JF438824 |
|  |  |  | LDA191 | NMVD73628 | NMVZ6226 | 9d | JF438112 | JF438349 | JF438587 | JF438825 |
|  |  |  | LDA192 | — | NMVZ10312 | 9d | JF438113 | JF438350 | JF438588 | JF438826 |
| 77 | Murrungowar Picnic Grd, Princes Hwy | VIC | LDA185 | — | NMVZ10310 | 9d | JF438105 | JF438342 | JF438580 | JF438818 |
|  |  |  | LDA186 | — | NMVZ10311 | 9d | JF438106 | JF438343 | JF438581 | JF438819 |
|  |  |  | LDA187 | NMVD73624 | NMVZ6222 | 9d | JF438107 | JF438344 | JF438582 | JF438820 |
|  |  |  | LDA188 | NMVD73625 | NMVZ6223 | 9d | JF438108 | JF438345 | JF438583 | JF438821 |
|  |  |  | LDA189 | NMVD73626 | NMVZ6224 | 9d | JF438109 | JF438346 | JF438584 | JF438822 |
| 78 | Buchan Caves Reserve, start of FJ Wilson Walk | VIC | LDA184 | NMVD73623 | NMVZ6221 | 9d | JF438104 | JF438341 | JF438579 | JF438817 |
| 79 | Western Port, The Gurdies | VIC | LDA102 | NMVD62031 | ABTC04041 | 9d | JF438019 | JF438257 | JF438494 | JF438732 |
| 80 | Eltham, Melbourne | VIC | LDA92 | SAMAR35541 | ABTC23330 | 9d | JF438239 | JF438476 | JF438714 | JF438952 |
|  |  |  | LDA95 | SAMAR35544 | ABTC23332 | 9d | JF438242 | JF438479 | JF438717 | JF438955 |
|  |  |  | LDA96 | SAMAR35542 | ABTC23331 | 9d | JF438243 | JF438480 | JF438718 | JF438956 |
| 81 | Main Yarra Trail, Yarra Flats, Ivanhoe, Melbourne | VIC | LDA124 | NMVD73631 | NMVZ6229 | 9d | JF438042 | HQ454791 | JF438517 | JF438755 |
| 82 | Little Desert NP | VIC | LDA13 | NMVD71552 | — | 7b | JF438048 | JF438285 | JF438523 | JF438761 |
|  |  |  | LDA14 | NMVD71552 | — | 7b | JF438059 | JF438296 | JF438534 | JF438772 |
| 83 | Greens Beach Coastal Trail, Tamar Valley | TAS | LDA198 | NMVD73636 | NMVZ6234 | 9d | JF438117 | JF438354 | JF438592 | JF438830 |
| 84 | Carr Villa Flora Reserve, Launceston | TAS | LDA199 | NMVD73637 | NMVZ6235 | 9d | JF438118 | JF438355 | JF438593 | JF438831 |
|  |  |  | LDA200 | NMVD73638 | NMVZ6236 | 9d | JF438120 | JF438357 | JF438595 | JF438833 |
|  |  |  | LDA201 | NMVD73639 | NMVZ6237 | 9d | JF438121 | JF438358 | JF438596 | JF438834 |
|  |  |  | LDA202 | NMVD73640 | NMVZ6238 | 9d | JF438122 | JF438359 | JF438597 | JF438835 |
| 85 | Ringarooma River Bridge, 1.1 km NE of Gladstone | TAS | LDA203 | — | NMVZ10316 | 9d | JF438123 | JF438360 | JF438598 | JF438836 |
|  |  |  | LDA204 | — | NMVZ10317 | 9d | JF438124 | JF438361 | JF438599 | JF438837 |
|  |  |  | LDA205 | — | NMVZ10318 | 9d | JF438125 | JF438362 | JF438600 | JF438838 |
|  |  |  | LDA206 | NMVD73641 | NMVZ6239 | 9d | JF438126 | JF438363 | JF438601 | JF438839 |
|  |  |  | LDA207 | — | NMVZ10319 | 9d | JF438127 | JF438364 | JF438602 | JF438840 |
| 86 | Ruby Creek bridge, South Mt Cameron township | TAS | LDA208 | NMVD73642 | NMVZ6240 | 9d | JF438128 | JF438365 | JF438603 | JF438841 |
|  |  |  | LDA209 | NMVD73643 | NMVZ6241 | 9d | JF438129 | JF438366 | JF438604 | JF438842 |
|  |  |  | LDA210 | — | NMVZ10320 | 9d | JF438131 | JF438368 | JF438606 | JF438844 |
| 87 | Lake Leake Rd | TAS | LDA94 | — | ABTC22990 | 9d | JF438241 | JF438478 | JF438716 | JF438954 |
| 88 | 12-24 km N Triabunna | TAS | LDA82 | — | ABTC23008 | 9d | JF438229 | JF438466 | JF438704 | JF438942 |
|  |  |  | LDA83 | — | ABTC23009 | 9d | JF438230 | JF438467 | JF438705 | JF438943 |
|  |  |  | LDA88 | — | ABTC22986 | 9d | JF438235 | JF438472 | JF438710 | JF438948 |
| 89 | Cascade Gardens, South Hobart | TAS | LDA211 | NMVD73644 | NMVZ6242 | 9d | JF438132 | JF438369 | JF438607 | JF438845 |
|  |  |  | LDA213 | NMVD73645 | NMVZ6243 | 9d | JF438133 | JF438370 | JF438608 | JF438846 |
| 90 | 1.5 km N Pine Hill HS | SA | LDA148 | SAMAR47049 | ABTC36220 | 7b | JF438068 | JF438305 | JF438543 | JF438781 |
| 91 | 13.5 km NW Frances | SA | LDA163 | SAMAR42272 | ABTC57639 | 7b | JF438085 | JF438322 | JF438560 | JF438798 |
|  |  |  | LDA171 | SAMAR42271 | ABTC57638 | 7b | JF438094 | JF438331 | JF438569 | JF438807 |
| 92 | 9.6 km WSW Binnum | SA | LDA179 | SAMAR49431 | ABTC37632 | 7b | JF438102 | JF438339 | JF438577 | JF438815 |
| 93 | 18.3 km N Coonawarra | SA | LDA166 | SAMAR49524 | ABTC37684 | 7b | JF438088 | JF438325 | JF438563 | JF438801 |
| 94 | 17.1 km WSW Straun | SA | LDA142 | SAMAR49483 | ABTC37495 | 7b | JF438062 | JF438299 | JF438537 | JF438775 |
|  |  |  | LDA146 | SAMAR49481 | ABTC37482 | 7b | JF438066 | JF438303 | JF438541 | JF438779 |
| 95 | Mary Seymour CP | SA | LDA126 | SAMAR26283 | ABTC68027 | 7b | JF438044 | JF438281 | JF438519 | JF438757 |
|  |  |  | LDA169 | SAMAR26284 | ABTC54337 | 7b | JF438091 | JF438328 | JF438566 | JF438804 |
|  |  |  | LDA170 | SAMAR26287 | ABTC54340 | 7b | JF438093 | JF438330 | JF438568 | JF438806 |
| 96 | 16 km ENE Greenways | SA | LDA165 | SAMAR49517 | ABTC37686 | 7b | JF438087 | JF438324 | JF438562 | JF438800 |
| 97 | 12.5 km SSW & 3.9 km E Lucindale | SA | LDA160 | SAMAR49526 | ABTC37694 | 7b | JF438082 | JF438319 | JF438557 | JF438795 |
|  |  |  | LDA167 | SAMAR49501 | ABTC37671 | 7b | JF438089 | JF438326 | JF438564 | JF438802 |
|  |  |  | LDA168 | SAMAR49508 | ABTC37670 | 7b | JF438090 | JF438327 | JF438565 | JF438803 |
| 98 | 3 km E & 3.4 km ENE Padthaway | SA | LDA155 | SAMAR49549 | ABTC37691 | 7b | JF438076 | JF438313 | JF438551 | JF438789 |
|  |  |  | LDA161 | SAMAR49550 | ABTC37693 | 7b | JF438083 | JF438320 | JF438558 | JF438796 |
|  |  |  | LDA164 | SAMAR49555 | ABTC37712 | 7b | JF438086 | JF438323 | JF438561 | JF438799 |
| 99 | 35 km ENE Kingston | SA | LDA150 | SAMAR47026 | ABTC36231 | 7b | JF438071 | JF438308 | JF438546 | JF438784 |
|  |  |  | LDA152 | SAMAR47037 | ABTC36242 | 7b | JF438073 | JF438310 | JF438548 | JF438786 |
| 100 | 25 km N Avenue | SA | LDA98 | SAMAR35818 | ABTC17006 | 7b | JF438245 | JF438482 | JF438720 | JF438958 |
|  |  |  | LDA99 | SAMAR35819 | ABTC17007 | 7b | JF438246 | JF438483 | JF438721 | JF438959 |
| 101 | 5.5-7.2 km SSW Bald Hill | SA | LDA129 | SAMAR53366 | ABTC70612 | 7b | JF438047 | JF438284 | JF438522 | JF438760 |
|  |  |  | LDA130 | SAMAR53364 | ABTC70607 | 7b | JF438049 | JF438286 | JF438524 | JF438762 |
| 102 | 0.5 km WSW Archie WH | SA | LDA172 | SAMAR49437 | ABTC37637 | 7b | JF438095 | JF438332 | JF438570 | JF438808 |
| 103 | 7.1 km ENE Abedour HS | SA | LDA157 | SAMAR49447 | ABTC37643 | 7b | JF438078 | JF438315 | JF438553 | JF438791 |
| 104 | 0.7 km NNE Mt Monster | SA | LDA176 | SAMAR49448 | ABTC37636 | 7b | JF438099 | JF438336 | JF438574 | JF438812 |
| 105 | 2.8 km NNE Duck Island HS | SA | LDA174 | SAMAR46067 | ABTC58325 | 7b | JF438097 | JF438334 | JF438572 | JF438810 |
| 106 | 4.2 km SSE Dunmore HS | SA | LDA162 | SAMAR45998 | ABTC58302 | 7b | JF438084 | JF438321 | JF438559 | JF438797 |
| 107 | 1.1-3.2 km ENE & 3.8 km SE Gum Lagoon | SA | LDA141 | SAMAR48657 | ABTC37084 | 7b | JF438061 | JF438298 | JF438536 | JF438774 |
|  |  |  | LDA143 | SAMAR48648 | ABTC37091 | 7b | JF438063 | JF438300 | JF438538 | JF438776 |
|  |  |  | LDA144 | SAMAR48660 | ABTC37092 | 7b | JF438064 | JF438301 | JF438539 | JF438777 |
| 108 | 4 km E Kendal HS, Bunbury Conservation Reserve | SA | LDA178 | SAMAR50255 | ABTC38062 | 7b | JF438101 | JF438338 | JF438576 | JF438814 |
| 109 | 14.3 km ENE Salt Creek | SA | LDA153 | SAMAR45160 | ABTC35076 | 7b | JF438074 | JF438311 | JF438549 | JF438787 |
|  |  |  | LDA154 | SAMAR45149 | ABTC35074 | 7b | JF438075 | JF438312 | JF438550 | JF438788 |
| 110 | 4.3 km WSW Salt Creek Trig | SA | LDA133 | SAMAR49268 | ABTC37400 | 7b | JF438052 | JF438289 | JF438527 | JF438765 |
|  |  |  | LDA136 | SAMAR49275 | ABTC37394 | 7b | JF438055 | JF438292 | JF438530 | JF438768 |
| 111 | 1 km NE Sunwood | SA | LDA134 | SAMAR39325 | ABTC34572 | 7b | JF438053 | JF438290 | JF438528 | JF438766 |
| 112 | 3 km SE Purple Downs | SA | LDA138 | SAMAR39670 | ABTC34717 | 7b | JF438057 | JF438294 | JF438532 | JF438770 |
| 113 | 6 km NW & 14km S Culburra | SA | LDA131 | SAMAR39656 | ABTC34695 | 7b | JF438050 | JF438287 | JF438525 | JF438763 |
|  |  |  | LDA139 | SAMAR39676 | ABTC34723 | 7b | JF438058 | JF438295 | JF438533 | JF438771 |
| 114 | 1 km SE Gum Flat | SA | LDA132 | SAMAR39406 | ABTC34687 | 7b | JF438051 | JF438288 | JF438526 | JF438764 |
|  |  |  | LDA135 | SAMAR39408 | ABTC34702 | 7b | JF438054 | JF438291 | JF438529 | JF438767 |
|  |  |  | LDA137 | SAMAR39409 | ABTC34714 | 7b | JF438056 | JF438293 | JF438531 | JF438769 |
| 115 | 7 km NW Nulungery | SA | LDA140 | SAMAR39405 | ABTC34386 | 7b | JF438060 | JF438297 | JF438535 | JF438773 |
| 116 | 3 km S Buccleuch | SA | LDA151 | SAMAR39602 | ABTC34663 | 7b | JF438072 | JF438309 | JF438547 | JF438785 |
| 117 | Bullock Hill Conservation Park | SA | LDA125 | SAMAR53396 | ABTC68906 | 7b | JF438043 | JF438280 | JF438518 | JF438756 |
|  |  |  | LDA127 | SAMAR53397 | ABTC68907 | 7b | JF438045 | JF438282 | JF438520 | JF438758 |
| 118 | Port Lincoln Area | SA | LDA67 | — | ABTC22928 | 7b | JF438215 | JF438452 | JF438690 | JF438928 |
| 119 | Tulka near Port Lincoln | SA | LDA177 | SAMAR51670 | ABTC58854 | 7b | JF438100 | JF438337 | JF438575 | JF438813 |
| 120 | 31 km WNW Coffin Bay Township | SA | LDA128 | SAMAR53551 | ABTC69270 | 7b | JF438046 | JF438283 | JF438521 | JF438759 |
