# Supplementary material for: Phylogeographic divergence in the widespread delicate skink (Lampropholis delicata) corresponds to dry habitat barriers in eastern Australia
Source: BMC Evol Biol. 2011 Jul 4;11:191. doi: 10.1186/1471-2148-11-191 (PMC3141439; doi:10.1186/1471-2148-11-191)
Supplement: Additional file 2 — Clades, haplotypes, latitude and longitude for Lampropholis delicata populations sampled in the study. [file 1471-2148-11-191-S2.DOC]

**Additional file 2** Collection localities of *Lampropholis delicata* samples used in the study. Population numbers and clades correspond to those listed in Figures 2,3,4,5. The distribution of the 165 haplotypes is indicated. The museum voucher specimen information and GenBank accession numbers are listed in Additional file 1. State abbreviations: QLD = Queensland; NSW = New South Wales; ACT = Australian Capital Territory; VIC = Victoria; TAS = Tasmania; SA = South Australia.

| **Population** | **Collection Locality** | **State** | **Latitude (°S)** | **Longitude (°E)** | **No. Samples** | **Clade** | **Haplotypes** |
| --- | --- | --- | --- | --- | --- | --- | --- |
| 1 | 6 km N The Crater turnoff on Kennedy | QLD | 17.40 | 145.52 | 1 | 1a | 1 |
| 2 | Railway estate, Townsville | QLD | 19.27 | 146.82 | 1 | 1a | 2 |
| 3 | Eungella | QLD | 21.12 | 148.48 | 1 | 1b | 3 |
| 4 | near Sarina | QLD | 21.44 | 149.17 | 2 | 1b | 4-5 |
| 5 | Shoalwater Bay | QLD | 22.35 | 150.39 | 1 | 1c | 6 |
| 6 | Shoalwater Bay Army reserve, N Rockhampton | QLD | 22.55 | 150.29 | 5 | 1c | 7-11 |
| 7 | Kidman Crt, Boyne Island | QLD | 23.95 | 151.35 | 1 | 1c | 12 |
| 8 | Bania SF | QLD | 25.00 | 151.49 | 2 | 1c | 13 |
| 9 | Blackdown Tableland | QLD | 23.79 | 149.10 | 4 | 6 | 14-15 |
| 10 | Kroombit Tops | QLD | 24.39 | 151.04 | 7 | 2 | 16-21 |
| 11 | Tablelands Rd, 5km E Amy's Peak, Kroombit Tops | QLD | 24.34 | 151.04 | 1 | 2 | 22 |
| 12 | Wongi SF | QLD | 25.42 | 152.53 | 1 | 3c | 23 |
| 13 | Tiaro, near Gympie | QLD | 25.73 | 152.61 | 1 | 3c | 24 |
| 14 | 25 km N Pomona | QLD | 26.19 | 152.91 | 1 | 3c | 25 |
| 15 | Lake Poonah, Cooloola NP | QLD | 26.21 | 153.03 | 4 | 3a | 26-29 |
| 16 | Bunya Mountains | QLD | 26.85 | 151.57 | 3 | 3b | 30 |
| 17 | Booloumba Creek, Conondale NP | QLD | 26.72 | 152.59 | 3 | 3c | 31-33 |
| 18 | Mt Nebo, D'Aguilar Range | QLD | 27.38 | 152.78 | 1 | 3c | 34 |
| 19 | The Gap, Brisbane | QLD | 27.44 | 152.94 | 3 | 3c | 34-35 |
| 20 | Parkinson, southern Brisbane | QLD | 27.65 | 153.03 | 1 | 3d | 36 |
| 21 | Barney View | QLD | 28.23 | 152.77 | 1 | 3d | 37 |
| 22 | Deongwar SF | QLD | 27.28 | 152.25 | 1 | 4a | 38 |
| 23 | Main Range | QLD | 28.10 | 152.42 | 1 | 4a | 39 |
| 24 | Python Rock, Lamington NP | QLD | 28.26 | 153.13 | 1 | 4a | 40 |
| 25 | Canungra Village, Lamington NP | QLD | 28.04 | 153.14 | 1 | 4a | 41 |
| 26 | Morans Falls, Lamington NP | QLD | 28.23 | 153.13 | 3 | 4a | 40, 42-43 |
| 27 | 50 km N Tenterfield, Wilsons Downfall Rd | NSW | 28.66 | 152.07 | 3 | 4a | 44-45 |
| 28 | Girard SF | NSW | 28.95 | 152.30 | 2 | 4a | 46-47 |
| 29 | Forestlands SF, trail off Spirabo FT ~5 km N Gurrs Rd | NSW | 29.35 | 152.06 | 2 | 4a | 48-49 |
| 30 | Mt Spirabo | NSW | 29.35 | 152.10 | 1 | 4a | 50 |
| 31 | 45 km E Glen Innes on Glen Innes-Grafton Rd | NSW | 29.63 | 152.14 | 2 | 4a | 51-52 |
| 32 | Chaelundi SF, Calicoe Creek, Liberation FT | NSW | 29.90 | 152.33 | 1 | 4a | 53 |
| 33 | Oakwood SF, Oakwood FT, N London Bridge FT | NSW | 29.90 | 152.09 | 3 | 4a | 54-55 |
| 34 | Glen Nevis SF, 3.3 km E Starlight Lookout | NSW | 29.92 | 152.21 | 2 | 4a | 56-57 |
| 35 | Riamukka SF, Grundy Fire Tower Area | NSW | 31.33 | 151.66 | 2 | 4a | 58 |
| 36 | Dorrigo NP, Never Never Picnic Area | NSW | 30.36 | 152.80 | 2 | 4a | 59-60 |
| 37 | Border Ranges NP, Tweed Valley Lookout | NSW | 28.37 | 153.10 | 3 | 4a,b | 61-62 |
| 38 | Border Ranges NP, Lophostemon Falls | NSW | 28.40 | 153.00 | 2 | 4b | 63-64 |
| 39 | Border Ranges NP, Pinnacle | NSW | 28.40 | 153.07 | 1 | 4b | 65 |
| 40 | Mount Warning NP | NSW | 28.40 | 153.30 | 4 | 4b | 66-69 |
| 41 | Nightcap NP, near Terania Creek Picnic Area | NSW | 28.60 | 153.30 | 2 | 4b | 70-71 |
| 42 | Whian Whian SF | NSW | 28.58 | 153.37 | 3 | 4b | 72-74 |
| 43 | Vict. Pk., S Alstonville | NSW | 28.90 | 153.37 | 1 | 4b | 75 |
| 44 | Torrington State Recreational Area, Torrington | NSW | 29.34 | 151.69 | 1 | 4c | 76 |
| 45 | Bolivia Hill | NSW | 29.33 | 151.90 | 2 | 4c | 77-78 |
| 46 | 18.5 km from Armidale on Ebor Rd | NSW | 30.53 | 151.86 | 2 | 4c | 79-80 |
| 47 | Yamba tip | NSW | 29.43 | 153.37 | 1 | 5a | 81 |
| 48 | Nana Creek Area, N Coffs Harbour | NSW | 30.18 | 152.96 | 3 | 5a | 82-83 |
| 49 | Glenreagh Area, N Coffs Harbour | NSW | 30.08 | 152.96 | 1 | 5a | 82 |
| 50 | Madman's Creek | NSW | 30.07 | 153.10 | 1 | 5a | 82 |
| 51 | Wedding Bells SF | NSW | 30.10 | 153.15 | 5 | 5a | 84-87 |
| 52 | Styx River SF, ~7 km on Styx Rd from Pt Lookout Rd | NSW | 30.50 | 152.30 | 3 | 5b | 88-89 |
| 53 | Werrikimbe NP, Plateau Beach | NSW | 31.18 | 152.32 | 2 | 5b | 90-91 |
| 54 | Cairncross SF | NSW | 31.38 | 152.60 | 2 | 5b | 92-93 |
| 55 | Coolah Tops NP, Rocky Creek Falls Picnic Area | NSW | 31.71 | 150.01 | 2 | 8 | 94 |
| 56 | Matthew Valley Rd, via Cooranbong | NSW | 33.08 | 151.45 | 1 | 9a | 95 |
| 57 | Doyalson | NSW | 33.20 | 151.50 | 1 | 9a | 96 |
| 58 | Warnervale Aerodome, N of Wyong | NSW | 33.24 | 151.43 | 3 | 9a | 97-99 |
| 59 | Homebush Bay, Cumbungi Wetland | NSW | 33.84 | 151.06 | 2 | 9a | 100-101 |
| 60 | University of Sydney | NSW | 33.89 | 151.08 | 5 | 9a | 102-103 |
| 61 | Padstow, Sydney | NSW | 33.96 | 151.03 | 1 | 9a | 103 |
| 62 | Coogee, Sydney | NSW | 33.93 | 151.25 | 3 | 9a | 104 |
| 63 | Hurstville, Sydney | NSW | 33.97 | 151.11 | 2 | 9a | 103 |
| 64 | Botany Bay NP | NSW | 33.98 | 151.24 | 2 | 9a | 105-106 |
| 65 | Cronulla Sewage Treatment Plant, Kurnell Peninsula | NSW | 34.03 | 151.16 | 3 | 9a | 107-109 |
| 66 | Royal NP | NSW | 34.13 | 151.05 | 1 | 9a | 110 |
| 67 | 3 km SW Brayton | NSW | 34.67 | 149.95 | 1 | 9b | 111 |
| 68 | Thirroul, N Side of Seafoam Ave, W of Railway Line | NSW | 34.30 | 150.90 | 1 | 9c | 112 |
| 69 | Rubbish tip, 4km NW Belmore Falls | NSW | 34.70 | 150.52 | 2 | 9c | 113-114 |
| 70 | Comerong Island | NSW | 34.88 | 150.74 | 6 | 9c | 115-119 |
| 71 | 2.6 km N Abercombie Rd crossing | NSW | 34.18 | 149.75 | 1 | 7a | 120 |
| 72 | Crace, Canberra | ACT | 35.22 | 149.13 | 1 | 7a | 121 |
| 73 | Birrigai | ACT | 35.45 | 148.95 | 1 | 7a | 122 |
| 74 | Princes Hwy, last rest stop VIC side VIC/NSW border | VIC | 37.38 | 149.67 | 1 | 9d | 123 |
| 75 | parkland near Genoa River Bridge, Genoa | VIC | 37.48 | 149.59 | 2 | 9d | 124-125 |
| 76 | Drummer Crk Picnic Grd, E Cann River, Princes Hwy | VIC | 37.57 | 149.27 | 3 | 9d | 126-127 |
| 77 | Murrungowar Picnic Grd, Princes Hwy | VIC | 37.68 | 148.62 | 5 | 9d | 127-129 |
| 78 | Buchan Caves Reserve, start of FJ Wilson Walk | VIC | 37.50 | 148.16 | 1 | 9d | 130 |
| 79 | Western Port, The Gurdies | VIC | 38.40 | 145.60 | 1 | 9d | 131 |
| 80 | Eltham, Melbourne | VIC | 37.72 | 145.15 | 3 | 9d | 132 |
| 81 | Main Yarra Trail, Yarra Flats, Ivanhoe, Melbourne | VIC | 37.77 | 145.07 | 1 | 9d | 133 |
| 82 | Little Desert NP | VIC | 36.50 | 141.80 | 2 | 7b | 140-141 |
| 83 | Greens Beach Coastal Trail, Tamar Valley | TAS | 41.09 | 146.74 | 1 | 9d | 132 |
| 84 | Carr Villa Flora Reserve, Launceston | TAS | 41.47 | 147.17 | 4 | 9d | 132, 134-135 |
| 85 | Ringarooma River Bridge, 1.1 km NE of Gladstone | TAS | 40.95 | 148.02 | 5 | 9d | 132, 136-137 |
| 86 | Ruby Creek bridge, South Mt Cameron township | TAS | 41.03 | 147.95 | 3 | 9d | 132, 138-139 |
| 87 | Lake Leake Rd | TAS | 41.99 | 147.68 | 1 | 9d | 132 |
| 88 | 12-24 km N Triabunna | TAS | 42.51 | 147.91 | 3 | 9d | 132, 136 |
| 89 | Cascade Gardens, South Hobart | TAS | 42.90 | 147.30 | 2 | 9d | 132 |
| 90 | 1.5 km N Pine Hill HS | SA | 36.26 | 140.94 | 1 | 7b | 142 |
| 91 | 13.5 km NW Frances | SA | 36.65 | 140.85 | 2 | 7b | 142-143 |
| 92 | 9.6 km WSW Binnum | SA | 36.83 | 140.83 | 1 | 7b | 144 |
| 93 | 18.3 km N Coonawarra | SA | 37.13 | 140.81 | 1 | 7b | 145 |
| 94 | 17.1 km WSW Straun | SA | 37.15 | 140.60 | 2 | 7b | 146-147 |
| 95 | Mary Seymour CP | SA | 37.17 | 140.62 | 3 | 7b | 146-147 |
| 96 | 16 km ENE Greenways | SA | 37.18 | 140.34 | 1 | 7b | 145, 148 |
| 97 | 12.5 km SSW & 3.9 km E Lucindale | SA | 37.07 | 140.29 | 3 | 7b | 142, 145 |
| 98 | 3 km E & 3.4 km ENE Padthaway | SA | 36.60 | 140.52 | 3 | 7b | 149-151 |
| 99 | 35 km ENE Kingston | SA | 36.76 | 140.26 | 2 | 7b | 142, 145 |
| 100 | 25 km N Avenue | SA | 36.75 | 140.22 | 2 | 7b | 142, 148 |
| 101 | 5.5-7.2 km SSW Bald Hill | SA | 36.58 | 140.02 | 2 | 7b | 152-153 |
| 102 | 0.5 km WSW Archie WH | SA | 36.40 | 140.29 | 1 | 7b | 142 |
| 103 | 7.1 km ENE Abedour HS | SA | 36.32 | 140.43 | 1 | 7b | 142 |
| 104 | 0.7 km NNE Mt Monster | SA | 36.20 | 140.32 | 1 | 7b | 142 |
| 105 | 2.8 km NNE Duck Island HS | SA | 36.22 | 140.13 | 1 | 7b | 154 |
| 106 | 4.2 km SSE Dunmore HS | SA | 36.27 | 140.04 | 1 | 7b | 142 |
| 107 | 1.1-3.2 km ENE & 3.8 km SE Gum Lagoon | SA | 36.27 | 139.98 | 3 | 7b | 142, 154-155 |
| 108 | 4 km E Kendal HS, Bunbury Conservation Reserve | SA | 36.14 | 139.93 | 1 | 7b | 156 |
| 109 | 14.3 km ENE Salt Creek | SA | 36.09 | 139.80 | 2 | 7b | 155, 157 |
| 110 | 4.3 km WSW Salt Creek Trig | SA | 36.14 | 139.72 | 2 | 7b | 142, 155 |
| 111 | 1 km NE Sunwood | SA | 35.94 | 139.69 | 1 | 7b | 154 |
| 112 | 3 km SE Purple Downs | SA | 35.88 | 140.22 | 1 | 7b | 158 |
| 113 | 6 km NW & 14km S Culburra | SA | 35.76 | 139.99 | 2 | 7b | 154, 159 |
| 114 | 1 km SE Gum Flat | SA | 35.71 | 140.10 | 3 | 7b | 154-155 |
| 115 | 7 km NW Nulungery | SA | 35.59 | 140.10 | 1 | 7b | 154 |
| 116 | 3 km S Buccleuch | SA | 35.37 | 139.88 | 1 | 7b | 160 |
| 117 | Bullock Hill Conservation Park | SA | 35.32 | 138.80 | 2 | 7b | 161,162 |
| 118 | Port Lincoln Area | SA | 34.72 | 135.86 | 1 | 7b | 163 |
| 119 | Tulka near Port Lincoln | SA | 34.80 | 135.80 | 1 | 7b | 164 |
| 120 | 31 km WNW Coffin Bay Township | SA | 34.53 | 135.16 | 1 | 7b | 165 |
