# Supplementary material for: Phylogeographic divergence in the widespread delicate skink (Lampropholis delicata) corresponds to dry habitat barriers in eastern Australia
Source: BMC Evol Biol. 2011 Jul 4;11:191. doi: 10.1186/1471-2148-11-191 (PMC3141439; doi:10.1186/1471-2148-11-191)
Supplement: Additional file 3 — Oligonucleotide primers used in this study. [file 1471-2148-11-191-S3.DOC]

**Additional file 3** Oligonucleotide primers used in this study. The letters L and H refer to the light and heavy strands. Values in ‘5’ position’ refer to the position of the 5’ position in the complete *Eumeces egregius* mtDNA sequence [1].

| Gene | Primer Name | Sequence (5’-3’) | 5’ Position | Source |
| --- | --- | --- | --- | --- |
| **ND2** | L4437 | AAGCTTTCGGGCCCATACC | 3833 | [2] |
|  | ND2r102 | CAGCCTAGGTGGGCGATTG | 4432 | [3] |
| **ND4** | ND4I | TGACTACCAAAAGCTCATGTAGAAGC | 10771 | [4] |
|  | tRNA-Leu | TACTTTTACTTGGATTTGCACCA | 11691 | [4] |
| ***12SrRNA*** | tPhe | AAAGCACRGCACTGAAGATGC | 23 | [5] |
|  | 12e | GTRCGCTTACCWTGTTACGACT | 982 | [5] |
| ***16SrRNA*** | L2510 | CGCCTGTTTATCAAAAACAT | 1907 | [6] |
|  | H3056 | CTCCGGTCTGAACTCAGATCACGTAGG | 2452 | Modified from [6] |

**References**

1. Kumazawa Y, Nishida M: **Complete mitochondrial DNA sequences of the green turtle and blue-tailed mole skink: statistical evidence for Archosaurian affinity of turtles**. *Molecular Biology and Evolution* 1999, **16**:784-792.
2. Macey JR, Larson A, Ananjeva NB, Fang Z, Papenfuss TJ: **Two novel gene orders and the role of light-strand replication in rearrangement of the vertebrate mitochondrial genome**. *Molecular Biology and Evolution* 1997, **14**:91-104.
3. Sadlier RA, Smith SA, Bauer AM, Whitaker AH: **A new genus and species of live-bearing scincid lizard (Reptilia: Scincidae) from New Caledonia**. *Journal of Herpetology* 2004, **38**:320-330.
4. Forstner MRJ, Davis SK, Arevalo E: **Support for the hypothesis of Anguimorph ancestry for the suborder Serpentes from phylogenetic analysis of mitochondrial DNA sequences**. *Molecular Phylogenetics and Evolution* 1995, **4**:93-102.
5. Reeder TW: **A phylogeny of the Australian *Sphenomorphus* group (Scincidae: Squamata) and the phylogenetic placement of the crocodile skinks (*Tribolonotus*): Bayesian approaches to assessing congruence and obtaining confidence in maximum likelihood inferred relationships**. *Molecular Phylogenetics and Evolution* 2003, **27**:384-397.
6. Palumbi SR: **Nucleic Acids II: The polymerase chain reaction**. In: *Molecular Systematics.* Edited by Hillis DM, Moritz C, Mable MK. Sunderland: Sinauer Associates; 1996: 205-247.
