# Supplementary material for: Phylogeographic divergence in the widespread delicate skink (Lampropholis delicata) corresponds to dry habitat barriers in eastern Australia
Source: BMC Evol Biol. 2011 Jul 4;11:191. doi: 10.1186/1471-2148-11-191 (PMC3141439; doi:10.1186/1471-2148-11-191)
Supplement: Additional file 5 — Divergence time estimates for the main Lampropholis delicata clades and subclades. [file 1471-2148-11-191-S5.DOC]

**Additional file 5** Divergence time estimates (million years) for the main *Lampropholis delicata* clades identified in Figures 4, 5 and 6. The mean, median and 95% confidence interval (highest posterior density, HPD) results from the BEAST analysis are presented.

| Clade | Mean | Median | 95% HPD interval |
| --- | --- | --- | --- |
| **Clade1** | 3.35 | 3.34 | 2.81-3.91 |
| Clade 1a | 0.99 | 0.98 | 0.68-1.32 |
| Clade 1b | 0.11 | 0.09 | 0.05-0.19 |
| Clade 1c | 0.26 | 0.26 | 0.14-0.40 |
| **Clade 2** | 0.17 | 0.17 | 0.08-0.29 |
| **Clade 3** | 2.34 | 2.33 | 1.97-2.73 |
| Clade 3a | 0.09 | 0.08 | 0.05-0.14 |
| Clade 3c | 0.72 | 0.71 | 0.51-0.93 |
| Clade 3d | 0.09 | 0.08 | 0.04-0.18 |
| **Clade 4** | 2.01 | 2.00 | 1.65-2.39 |
| Clade 4a | 1.03 | 1.02 | 0.79-1.30 |
| Clade 4b | 0.26 | 0.25 | 0.14-0.39 |
| Clade 4c | 1.11 | 1.10 | 0.80-1.44 |
| **Clade 5** | 1.61 | 1.59 | 1.23-2.01 |
| Clade 5a | 0.35 | 0.35 | 0.19-0.53 |
| Clade 5b | 0.56 | 0.55 | 0.37-0.76 |
| **Clade 6** | 0.06 | 0.06 | 0.02-0.11 |
| **Clade 7** | 2.13 | 2.12 | 1.68-2.55 |
| Clade 7a | 0.12 | 0.11 | 0.05-0.22 |
| Clade 7b | 0.59 | 0.58 | 0.41-0.77 |
| **Clade 9** | 2.76 | 2.75 | 2.31-3.21 |
| Clade 9a | 0.68 | 0.67 | 0.46-0.92 |
| Clade 9c | 0.22 | 0.21 | 0.10-0.34 |
| Clade 9d | 0.27 | 0.26 | 0.15-0.39 |
| ***L. delicata* lineage** | 6.01 | 5.99 | 5.30-6.74 |
| **Tree root** | 19.77 | 19.84 | 19.32-20.00 |
